# Model Decoupled Synchronization Control Design with Fractional Order Filter for H-Type Air Floating Motion Platform

**DOI:** 10.3390/e23050633

**Published:** 2021-05-19

**Authors:** Yixiu Sun, Lizhan Zeng, Ying Luo, Xiaoqing Li

**Affiliations:** School of Mechanical Science and Engineering, Huazhong University of Science and Technology, Wuhan 430070, China; sunyx@hust.edu.cn (Y.S.); zenglizhan@hust.edu.cn (L.Z.); ying.luo@hust.edu.cn (Y.L.)

**Keywords:** H-type linear motor air floating motion platform, Dynamic modeling, Synchronous control, Feed-forward control, Fractional order biquad filter

## Abstract

H-type motion platform with linear motors is widely used in two-degrees-of-freedom motion systems, and one-direction dual motors need to be precisely controlled with strict synchronization for high precision performance. In this paper, a synchronous control method based on model decoupling is proposed. The dynamic model of an H-type air floating motion platform is established and one direction control using two motors with position dependency coupling is decoupled and converted into independent position and rotation controls, separately. For the low damping second-order oscillation system of the rotation control loop, a new fractional order biquad filtering method is proposed to generate an antiresonance peak to improve the phase and control gain of the open loop system, which can ensure system stability and quick attenuation for external disturbances. In the multiple-degree-of-freedom decoupled control loops, a systematic feedback controller design methodology is proposed to satisfy the given frequency domain design specifications; a feed-forward control strategy is also applied to compensate the disturbance torque caused by the platform motion. The simulation and experimental results demonstrate that the proposed synchronization control method is effective, and achieves better disturbance rejection performance than the existing optimal cancellation filtering method and biquad filtering method.

## 1. Introduction

H-type air floating motion platform can realize two degrees of freedom large stroke precise positioning motion and is widely applied in lithography silicon wafer stage, precision measuring equipment, and laser drilling equipment [1,2,3]. The platform is directly driven by the linear motor and supported and guided by the air floating guideway, which has the advantages of symmetrical structure and large overall stiffness. One direction of H-type motion platform is driven by a single motor, and the other direction is driven by double motors, which can provide larger driving force. The position of the double motors needs to be precisely synchronously controlled, otherwise, the positioning accuracy in two degrees of freedom cannot be guaranteed, and the poor synchronization accuracy will lead to a stuck guide rail.

There are several existing synchronization control methods. The parallel control method is to build two independent control loops with the same reference position, and the synchronization accuracy of the dual motors is determined by the position tracking accuracy of the two loops [4,5]. In the master-slave control method, there are two independent master and slave loops. The feedback position of the master loop is taken as the reference position of the slave loop, and the synchronization accuracy is determined by the control performance of the slave loop [6]. In the cross-coupling control method, a synchronous error controller is introduced into the parallel control, which generates control signals with the position difference of the dual motors to realize synchronous control [7,8,9,10,11]. The disturbance observer is introduced into the loop to realize synchronous control [12,13]. The Lagrangian dynamic model of the H-type motion platform is established, which converts the position of the double motors into the position and angle around the middle point of the beam for control [14]. In these control methods, the position coupling between the dual motors connected by the beam of the H-type motion platform is not considered, the controller is difficult to design, and the system stability cannot be guaranteed [15]. To solve this problem, a control method based on coordinate decoupling is proposed. According to the dynamic model of the H-type motion platform, the position of the two motors is converted into the position and rotation angle at the centroid of the entire component to realize the position tracking and synchronous error control. The two degrees of freedom of position and rotation are naturally decoupled, which is conducive to designing the controller. This decoupling method is used to control the position of the dual motor driven linear slider, but no moving part is on the beam [15]. The feed-forward control is introduced to improve the position tracking accuracy based on the decoupled model [16], but the research on introducing feed-forward control into the rotation control loop to improve the synchronization accuracy is scarce. Sliding mode control is introduced into the decoupled three-degrees-of-freedom model to realize the precise contour control of the platform [17]. For the platform with moving parts, the thrust of the double motors is distributed to control the position at the centroid of the entire component [18]. The movement of the components on the beam is considered, and the adaptive method is adopted to realize the position and angle control [19,20,21].

The rotation model of H-type air floating motion platform is a second-order oscillation system with low damping. There is a resonance peak in the amplitude-frequency characteristic curve, and the corresponding phase decreases rapidly from 0° to −180°. The control gain needs to be designed very small to make the gain margin of the system greater than 0, but the control performance cannot be guaranteed. For the low damping oscillation system, the tuned mass damping module can be used to increase the damping of the system [22], so as to attenuate the resonance peak and reduce the phase change speed. The mass, stiffness, and damping of the module need to be designed according to the original system parameters. For the system with low frequency mechanical resonance, there are resonance and antiresonance peaks in the Bode diagram of open-loop transfer function. The low pass filter, notch filter, and biquad filter are applied to reduce the amplitude at the resonance peak to improve the gain margin of the system [23]. The matched biquad filter can completely eliminate the resonance and antiresonance peaks, but it is sensitive to the changes of plant parameters and the disturbance rejection performance is poor. For the low damping oscillation system, it is difficult to guarantee the tracking performance and disturbance rejection performance of the closed-loop system simultaneously.

In this paper, a synchronous control method based on coordinate decoupling with feedback and feed-forward control is proposed for H-type motion platform, and a fractional order biquad filter is introduced into the low damping second-order oscillation system to achieve good tracking and disturbance rejection performance simultaneously.

The main contributions of this paper are as follows, (1) The X, Y, and Rz directions dynamic models of H-type air floating motion platform are established, and a synchronous control method based on coordinate decoupling is proposed, which transforms the position control of the direction with two motors into X position and Rz rotation control, and the Rz torque feed-forward control based on the established model is also applied to reduce the synchronization error caused by the platform motion. (2) For the low damping second-order oscillation system in Rz rotation control, a fractional order biquad filter with antiresonance peak is proposed, which can ensure the desired tracking and disturbance rejection performance of the system, simultaneously. (3) A systematic feedback control design method with the given design specifications are proposed for the X, Y, and Rz control loops. The optimal order of the fractional order biquad filter is obtained by minimizing the peak value of the process sensitive transfer function. Simulation and experimental results show that the proposed control method is effective and can achieve better disturbance rejection performance than the existing optimal cancellation filtering method and biquad filtering method. The difference between this paper and the previous work are: (1) after decoupling modeling of the air bearing platform, this paper points out the low damping oscillation characteristics of the rotation control loop, and the existence of the resonance peak, makes the controller difficult to design. (2) Compared with the cancellation filtering method, a new non-cancellation filtering method is proposed to realize the synchronization control, and a fractional order filter is introduced to improve the disturbance rejection performance of the system. (3) Compared with the existing synchronous feedback control method, the feed-forward control is added to compensate for the synchronous disturbance torque caused by the platform motion.

The paper is organized as follows. In Section 2, the dynamic model of the H-type air floating motion platform is established, and the Rz disturbance torque caused by the platform motion is modeled. Then the synchronous control method based on model decoupling is proposed, the feedback and feed-forward controllers in X, Y, and Rz control loops are designed. In Section 3, the proposed synchronization control method is verified in simulation and experiment. Conclusions are drawn in Section 4.

## 2. Materials and Methods

### 2.1. Dynamic Modeling of H-Type Air Floating Motion Platform

**Figure 1:**
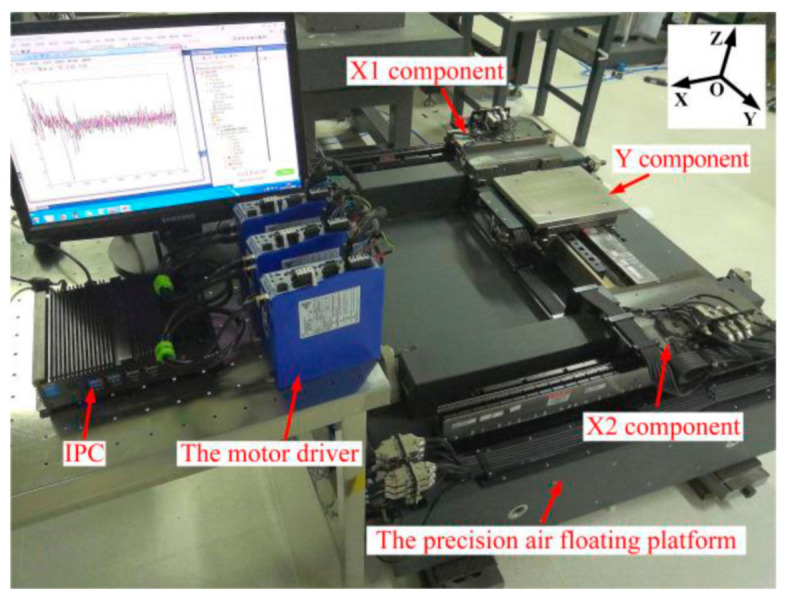
H-type air floating motion platform.

An H-type air floating motion platform is shown in Figure 1. The platform is driven by two motors in X-direction, and the positions of the X1 and X2 components are measured by two grating rulers, seperately. X1 and X2 components are connected by the beam, and the Y component is sheathed on the outside of the beam to realize the X-direction movement. The Y component is driven by a single linear motor, and the grating ruler measures the position to realize the Y-direction movement. In the X and Y directions, the air floating guideway is used for supporting and guiding, which eliminates the friction and provides the vertical and horizontal stiffness and damping to ensure the overall structural stiffness of the system. In order to control the platform, it is necessary to obtain the transfer function of the control plant from the control force to the feedback position. Next, the dynamic model of the platform is established.

**Figure 2:**
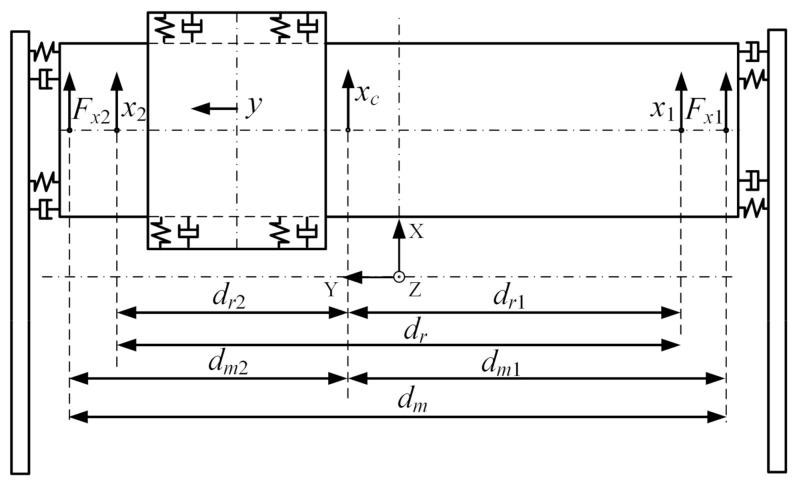
The structure diagram of H-type motion platform.

The schematic diagram of H-type motion platform is shown in Figure 2, the XY coordinate system takes the midpoint of the stroke as the origin, and the system parameters are shown in Table 1.

#### 2.1.1. Y-direction Dynamic Model

**Figure 3:**
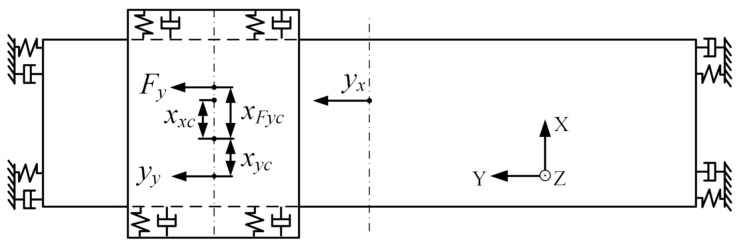
Y component moves along Y-direction.

As shown in Figure 3, the Y component moves along the Y-direction under the Y motor force $F_{y}$, and its absolute position is $y_{y}$. The X component moves along the Y-direction under the Y motor stator reaction force $-F_{y}$, and its absolute position is $y_{x}$.

The dynamic equation is:

(1) $$m_{y}{\ddot{y}}_{y}\left(t\right)=F_{y}\left(t\right)=K_{fy}\cdot i_{y}\left(t-\tau \right),$$

(2) $$m_{x}{\ddot{y}}_{x}\left(t\right)+4c_{xH}{\dot{y}}_{x}\left(t\right)+4k_{xH}y_{x}\left(t\right)=-F_{y}\left(t\right).$$

Since the feedback position y is the position of the Y component relative to the beam, i.e., $y=y_{y}-y_{x}$, the transfer function between y and Y motor current $i_{y}$ is obtained:

(3) $$\frac{y\left(s\right)}{i_{y}\left(s\right)}=\frac{K_{fy}}{m_{y}s^{2}}\frac{\left(m_{x}+m_{y}\right)s^{2}+4c_{xH}s+4k_{xH}}{m_{x}s^{2}+4c_{xH}s+4k_{xH}}e^{-\tau s}.$$

#### 2.1.2. X-direction Dynamic Model

**Figure 4:**
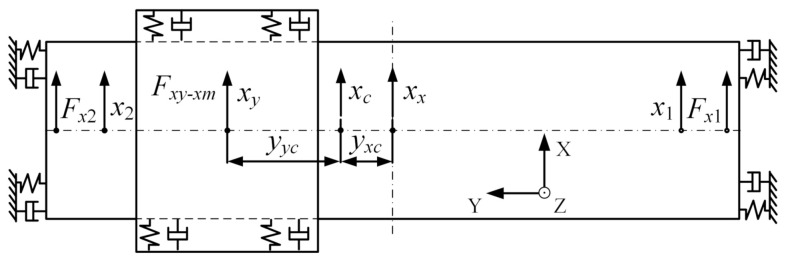
The platform moves along X-direction.

As shown in Figure 4, the entire moving components move along X-direction under the X motors force, and the position of X component is $x_{x}$. The Y component moves under the Y horizontal air floating force $F_{xy-xm}$, and its position is $x_{y}$.

The dynamic equation is:

(4) $$m_{y}{\ddot{x}}_{y}\left(t\right)=F_{xy-xm}\left(t\right),$$

(5) $$m_{x}{\ddot{x}}_{x}\left(t\right)=F_{x}\left(t\right)+F_{yx-xm}\left(t\right),$$

where, $F_{xy-xm}\left(t\right)=4c_{yH}\left({\dot{x}}_{x}\left(t\right)-{\dot{x}}_{y}\left(t\right)\right)+4k_{yH}\left(x_{x}\left(t\right)-x_{y}\left(t\right)\right)$, $F_{yx-xm}$ is the air floating reaction force of Y component on beam. $F_{x_{1}}$ is the X1 motor force, $F_{x_{2}}$ is the X2 motor force, and $F_{x}$ is the total force of the two motors,

(6) $$F_{x}\left(t\right)=F_{x_{1}}\left(t\right)+F_{x_{2}}\left(t\right)=K_{fx_{1}}\cdot i_{x_{1}}\left(t-\tau \right)+K_{fx_{2}}\cdot i_{x_{2}}\left(t-\tau \right).$$

Since the rotation angle of the entire moving component around the Z-axis is small, the position $x_{x}$ at the centroid of the X component is considered the same as the position $x_{c}$ at the centroid of the entire moving component, that is, $x_{x}\approx x_{c}$. The relationship between $x_{c}$ and the measured position $x_{1}$, $x_{2}$ is as follows,

(7) $$x_{c}\left(t\right)=\frac{d_{r2}\left(t\right)}{d_{r}}x_{1}\left(t\right)+\frac{d_{r1}\left(t\right)}{d_{r}}x_{2}\left(t\right).$$

The transfer function between $x_{c}$ and $F_{x}$ is obtained from Equations (4) and (5),

(8) $$\frac{x_{c}\left(s\right)}{F_{x}\left(s\right)}=\frac{1}{d_{r}}\frac{d_{r2}x_{1}\left(s\right)+d_{r1}x_{2}\left(s\right)}{K_{fx_{1}}\cdot i_{x_{1}}\left(s\right)e^{-\tau s}+K_{fx_{2}}\cdot i_{x_{2}}\left(s\right)e^{-\tau s}}=\frac{1}{\left(m_{x}+m_{y}\right)s^{2}}\frac{m_{y}s^{2}+4c_{yH}s+4k_{yH}}{\frac{m_{x}m_{y}}{m_{x}+m_{y}}s^{2}+4c_{yH}s+4k_{yH}}.$$

#### 2.1.3. Rz Dynamic Model

According to Figure 4, the entire moving component will rotate around the Z-axis at the centroid under the X1 and X2 motors force.

(9) $$J_{Yz}{\ddot{\theta }}_{yz}\left(t\right)=T_{xy-xr}\left(t\right),$$

(10) $$\begin{array}{c} J_{Xz}{\ddot{\theta }}_{xz}\left(t\right)+c_{xH}d_{x}H^{2}{\dot{\theta }}_{xz}\left(t\right)+k_{xH}d_{x}H^{2}\theta_{xz}\left(t\right)=T_{\theta }\left(t\right)+T_{yx-xr}\left(t\right) \end{array},$$

where, $T_{xy-xr}$ is the torque produced by the rotation of X component relative to Y component, $T_{xy-xr}\left(t\right)=c_{yH}d_{y}H^{2}\left({\dot{\theta }}_{xz}\left(t\right)-{\dot{\theta }}_{yz}\left(t\right)\right)+k_{yH}d_{yH^{2}}\left(\theta_{xz}\left(t\right)-\theta_{yz}\left(t\right)\right)$, and $T_{yx-xr}$ is the reaction torque. $T_{\theta }$ is the torque of the X1 and X2 motors force on the centroid:

(11) $$T_{\theta }\left(t\right)=K_{fx_{1}}\cdot i_{x_{1}}\left(t-\tau \right)\cdot d_{m1}\left(t\right)-K_{fx_{2}}\cdot i_{x_{2}}\left(t-\tau \right)\cdot d_{m2}\left(t\right),$$

Although $d_{m1}$, $d_{m2}$ will change with the movement of Y component, they are not in the control loop and will not be affected by the system delay. $\theta_{xz}$ can be regarded as the angle of the entire moving component around Z-axis, that is, $\theta_{z}\approx \theta_{xz}$. $\theta_{z}$ can be calculated by the two measured positions $x_{1}$ and $x_{2}$,

(12) $$\theta_{z}\left(t\right)=\frac{x_{1}\left(t\right)-x_{2}\left(t\right)}{d_{r}}.$$

Since $J_{Yz}$ is smaller than $J_{Xz}$ in the experimental system, $J_{Yz}{}^{2}$ can be approximately 0 in the simplified calculation. The transfer function between $\theta_{z}$ and $T_{\theta }$ can be obtained as follows,

(13) $$\begin{array}{ll} \frac{\theta_{z}\left(s\right)}{T_{\theta }\left(s\right)} & =\frac{1}{d_{r}}\frac{x_{1}\left(s\right)-x_{2}\left(s\right)}{K_{fx_{1}}\cdot i_{x_{1}}\left(s\right)e^{-\tau s}\cdot d_{m1}\left(s\right)-K_{fx_{2}}\cdot i_{x_{2}}\left(s\right)e^{-\tau s}\cdot d_{m2}\left(s\right)}\\ & =\frac{J_{Yz}s^{2}+c_{yH}d_{yH^{2}}s+k_{yH}d_{yH^{2}}}{[\begin{array}{c} \left(J_{Xz}s^{2}+c_{xH}d_{x}H^{2}s+k_{xH}d_{x}H^{2}\right)\left(J_{Yz}s^{2}+c_{yH}d_{y}H^{2}s+k_{yH}d_{y}H^{2}\right)\\ +J_{Yz}s^{2}(c_{yH}d_{y}H^{2}s+ky_{H}d_{y}H^{2}) \end{array}]}\\ & =\frac{J_{Yz}s^{2}+c_{yH}d_{y}H^{2}s+k_{yH}d_{y}H^{2}}{\left[\begin{array}{c} \left(J_{Xz}s^{2}+J_{Yz}s^{2}+c_{xH}d_{x}H^{2}s+k_{xH}d_{x}H^{2}\right)\left(J_{Yz}s^{2}+c_{yH}d_{y}H^{2}s+k_{yH}d_{y}H^{2}\right)\\ -J_{Yz}s^{2}J_{Yz}s^{2} \end{array}\right]}\\ & \approx \frac{1}{J_{Xz}s^{2}+J_{Yz}s^{2}+c_{xH}d_{x}H^{2}s+k_{xH}d_{x}H^{2}} \end{array}$$

#### 2.1.4. The Disturbance Torque Model in Rz from X Moves

According to Figure 4, when the platform moves along the X-direction and the Y component is not located in the middle of the beam, the air floating reaction force $-F_{xy-xm}$ of the Y component on the beam and the inertia force of the X component do not pass through the centroid of the entire component, resulting in the disturbance torque in Rz. Assuming that the disturbance torque is $T_{d-xm}$, the opposite driving torque $-T_{d-xm}$ is introduced into the control loop to act on the X component to remain the angle of the X component at 0. When the X component does not rotate, the torque of beam to Y component is 0 and Y component will not rotate. The dynamic equation of X component is:

(14) $$-T_{d-xm}\left(t\right)+T_{x1-xm}\left(t\right)+T_{x2-xm}\left(t\right)=0,$$

where, $T_{x1-xm}$ is the torque of inertia force of X component on the centroid of the entire component, $T_{x1-xm}\left(t\right)=m_{x}{\ddot{x}}_{x}\left(t\right)\cdot y_{xc}\left(t\right)$. $T_{x2-xm}$ is the torque of $-F_{xy-xm}$ on the centroid of the entire component. Then, the disturbance torque is obtained as,

(15) $$\theta_{z}\left(t\right)=\frac{x_{1}\left(t\right)-x_{2}\left(t\right)}{d_{r}}.$$

#### 2.1.5. The Disturbance Torque Model in Rz from Y Moves

According to Figure 3, when the Y component moves along the Y-direction, the eccentric drive in X-direction will produce the disturbance torque in Rz. Assuming that the disturbance torque is $T_{d-ym}$, the opposite driving torque $-T_{d-ym}$ is introduced into the control loop to act on the X component to remain the angle of the X component at 0. The dynamic equation of Y component is

(16) $$\begin{array}{c} J_{Yz}{\ddot{\theta }}_{yz}\left(t\right)=T_{y1-ym}\left(t\right)+T_{y2-ym}\left(t\right)+T_{y3-ym}\left(t\right) \end{array},$$

where, $T_{y1-ym}$ is the torque of Y motor force on the centroid of the entire component, $T_{y1-ym}\left(t\right)=F_{y}\left(t\right)x_{F_{y}c}$. $T_{y2-ym}$ is the torque of the inertia force of Y component on the centroid of the entire component, $T_{y2-ym}\left(t\right)=-m_{y}{\ddot{y}}_{y}\left(t\right)x_{yc}$. $T_{y3-ym}$ is the air floating torque of the beam to Y component, $T_{y3-ym}\left(t\right)=c_{yH}d_{y}H^{2}\left({\dot{\theta }}_{xz}\left(t\right)-{\dot{\theta }}_{yz}\left(t\right)\right)+k_{yH}d_{y}H^{2}\left(\theta_{xz}\left(t\right)-\theta_{yz}\left(t\right)\right)$. Since $\theta_{xz}\left(t\right)=0$, then $T_{y3-ym}\left(t\right)=-c_{yH}d_{y}H^{2}{\dot{\theta }}_{yz}\left(t\right)-k_{yH}d_{y}H^{2}\theta_{yz}\left(t\right)$.

After introducing $-T_{d-ym}$, the angle of the X component is 0, the dynamic equation of X component is:

(17) $$\begin{array}{c} -T_{d-ym}\left(t\right)+T_{x1-ym}\left(t\right)+T_{x2-ym}\left(t\right)+T_{x3-ym}\left(t\right)=0 \end{array},$$

where $T_{x1-ym}$ is the torque of the Y motor stator reaction force on the centroid of the entire component, $T_{x1-ym}\left(t\right)=-F_{y}\left(t\right)x_{F_{y}c}$. $\;T_{x2-ym}$ is the torque of inertia force of X component on the centroid of the entire component, $T_{x2-ym}\left(t\right)=m_{x}{\ddot{y}}_{x}\left(t\right)x_{xc}=-\frac{F_{y}\left(t\right)m_{x}x_{xc}s^{2}}{m_{x}s^{2}+4c_{xH}s+4k_{xH}}$. $T_{x3-ym}$ is the air floating torque of Y component to the beam, $T_{x3-ym}\left(t\right)=-T_{y3-ym}\left(t\right)$. Then the disturbance torque is obtained as,

(18) $$\begin{array}{ll} T_{d-ym}\left(s\right)=-F_{y}\left(s\right)( & \frac{m_{x}s^{2}}{m_{x}s^{2}+4c_{xH}s+4k_{xH}}x_{xc}+\frac{J_{Yz}s^{2}}{J_{Yz}s^{2}+c_{yH}d_{y}H^{2}s+k_{yH}d_{y}H^{2}}x_{F_{y}c}+\\ & \frac{c_{yH}d_{y}H^{2}s+k_{yH}d_{y}H^{2}}{J_{Yz}s^{2}+c_{yH}d_{y}H^{2}s+k_{yH}d_{y}H^{2}}x_{yc}) \end{array}.$$

### 2.2. Synchronous Control Method Based on Model Decoupling

The Y and X directions of H-type motion platform are naturally decoupled and can be controlled separately. The Y control plant is the transfer function from Y feedback position to Y control current, as shown in Equation (3).

In the X and Rz direction dynamic modeling, it can be seen from Equations (8) and (13) that the two measured positions $x_{1}$ and $x_{2}$ are coupled with each other due to the beam connection. If $x_{1}$ and $x_{2}$ are controlled directly, the transfer functions from $i_{x_{1}}$ to $x_{1}$ and from $i_{x_{2}}$ to $x_{2}$ can not be obtained, and the controller is difficult to design. Since the X position $x_{c}$ and the Rz angle $\theta_{z}$ at the centroid are two naturally decoupled and independent degrees of freedom, this paper transforms the two positions $x_{1}$ and $x_{2}$ into the position $x_{c}$ and the angle $\theta_{z}$ for control. It can be seen from Equations (8) and (13) that the transfer functions from $x_{c}$ to $F_{x}$ and from $\theta_{z}$ to $T_{\theta }$ are clear. Based on these, two controllers can be designed to realize the X position control and the Rz rotation control, and the reference angle in Rz loop is 0 to realize the synchronous motion control of the dual motors.

In order to realize the position and rotation control, the X thrust constant $K_{fx}$ and the Rz thrust constant $K_{f\theta }$ are established, and design $K_{fx}=K_{f\theta }=\frac{K_{fx_{1}}+K_{fx_{2}}}{2}$. According to Equations (6) and (11), the X force $F_{x}$ and Rz torque $T_{\theta }$ are:

(19) $$F_{x}\left(t\right)=K_{fx_{1}}\cdot i_{x_{1}}\left(t-\tau \right)+K_{fx_{2}}\cdot i_{x_{2}}\left(t-\tau \right)=K_{fx}\cdot i_{x}\left(t-\tau \right),$$

(20) $$\begin{array}{c} T_{\theta }\left(t\right)=K_{fx_{1}}\cdot i_{x_{1}}\left(t-\tau \right)\cdot d_{m1}\left(t\right)-K_{fx_{2}}\cdot i_{x_{2}}\left(t-\tau \right)\cdot d_{m2}\left(t\right)=K_{f\theta }\cdot i_{\theta }\left(t-\tau \right) \end{array}.$$

The position control signal $i_{x}$ and rotation control signal $i_{\theta }$ need to be converted into the control signals $i_{x_{1}}$ and $i_{x_{2}}$ to drive the dual motors.

(21) $$\left[\begin{array}{c} i_{x_{1}}\left(t\right)\\ i_{x_{2}}\left(t\right) \end{array}\right]=\frac{1}{d_{m}}\left[\begin{array}{cc} \frac{K_{fx}\cdot d_{m2}\left(t+\tau \right)}{K_{fx_{1}}} & \frac{K_{f\theta }}{K_{fx_{1}}}\\ \frac{K_{fx}\cdot d_{m1}\left(t+\tau \right)}{K_{fx_{2}}} & -\frac{K_{f\theta }}{K_{fx_{2}}} \end{array}\right]\left[\begin{array}{c} i_{x}\\ i_{\theta } \end{array}\right],$$

where the time-varying signals $d_{m1}$ and $d_{m2}$ are advanced to compensate the delay effect, so as to ensure that the resultant force of the two motors can pass through the centroid of the entire component when the Y component moves.

In order to realize the closed-loop control, the two measured positions $x_{1}$ and $x_{2}$ need to be converted into the position $x_{c}$ and the angle $\theta_{z}$ and fed back to the two control loops, respectively. According to Equations (7) and (12), the conversion between $x_{c}$, $\theta_{z}$ and $x_{1}$, $x_{2}$ can be obtained as

(22) $$\left[\begin{array}{c} x_{c}\left(t\right)\\ \theta_{z}\left(t\right) \end{array}\right]=\frac{1}{d_{r}}\left[\begin{array}{cc} d_{r2}\left(t\right) & d_{r1}\left(t\right)\\ 1 & -1 \end{array}\right]\left[\begin{array}{c} x_{1}\left(t\right)\\ x_{2}\left(t\right) \end{array}\right].$$

According to Equations (8), (13) and (19), (20), the X and Rz control plants are obtained as

(23) $$P_{x}\left(s\right)=\frac{x_{c}\left(s\right)}{i_{x}\left(s\right)}=\frac{K_{fx}}{\left(m_{x}+m_{y}\right)s^{2}}\frac{m_{y}s^{2}+4c_{yH}s+4k_{yH}}{\frac{m_{x}m_{y}}{m_{x}+m_{y}}s^{2}+4c_{yH}s+4k_{yH}}e^{-\tau s},$$

(24) $$P_{\theta }\left(s\right)=\frac{\theta_{z}\left(s\right)}{i_{\theta }\left(s\right)}=\frac{K_{f\theta }}{\left(J_{Xz}+J_{Yz}\right)s^{2}+c_{xH}d_{x}H^{2}s+k_{xH}d_{x}H^{2}}.$$

**Figure 5:**
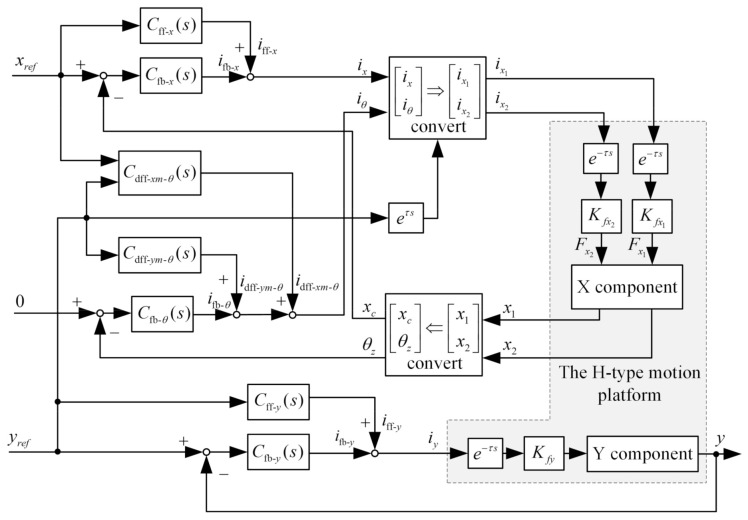
The block diagram of the platform control system.

The control system of the H-type motion platform is established as Figure 5.

#### 2.2.1. Rz Controller Design

##### Rz Control Plant

It can be seen from Equation (24) that the Rz control plant is a second-order oscillation system with time delay and it can be transformed into the following equation,

(25) $$\begin{array}{ll} P_{\theta }\left(s\right) & =\frac{\theta_{z}\left(s\right)}{i_{\theta }\left(s\right)}=\frac{K_{f\theta }}{J_{z}s^{2}+c_{xH}d_{x}H^{2}s+k_{xH}d_{x}H^{2}}e^{-\tau s}\\ & =\frac{K_{f\theta }}{k_{xH}d_{x}H^{2}}\frac{{\left(2\pi f_{n-\theta }\right)}^{2}}{s^{2}+2\zeta_{\theta }2\pi f_{n-\theta }s+{\left(2\pi f_{n-\theta }\right)}^{2}}e^{-\tau s} \end{array},$$

where, $J_{z}=J_{Xz}+J_{Yz}=J_{Xz0}+J_{Yz0}+\frac{m_{x}m_{y}}{m_{x}+m_{y}}y^{2}$, $f_{n-\theta }=\frac{1}{2\pi }\sqrt{\frac{k_{xH}d_{x}H^{2}}{J_{z}}}$, $\zeta_{\theta }=\frac{c_{xH}d_{xH}}{2\sqrt{J_{z}k_{xH}}}$. Due to the low damping of the air floating guideway, there is a resonance peak in its amplitude-frequency curve, and the phase-frequency curve decreases 180° rapidly near the natural frequency, then the phase-frequency curve of the open-loop transfer function is prone to have a −180° phase crossover point. The controller gain should be designed small enough to ensure that the peak is below 0 dB and the system has gain margin. In the existing literature, the biquad filter is introduced to eliminate the peaks and troughs in the open-loop transfer function, which makes the open-loop transfer function smooth, but the disturbance rejection performance of the system is very poor [23].

A fractional biquad filter [24] is introduced into the system and its transfer function is:

(26) $$C_{\mathrm{biq}-\theta }=\frac{f_{n2-\mathrm{biq}-\theta }{}^{2}}{f_{n1-\mathrm{biq}-\theta }{}^{2}}\frac{s^{2}+2\zeta_{1-\mathrm{biq}-\theta }2\pi f_{n1-\mathrm{biq}-\theta }s+{\left(2\pi f_{n1-\mathrm{biq}-\theta }\right)}^{2}}{s^{2}+2\zeta_{2-\mathrm{biq}-\theta }2\pi f_{n2-\mathrm{biq}-\theta }s^{r}+{\left(2\pi f_{n2-\mathrm{biq}-\theta }\right)}^{2}}.$$

The difference between this filter and the biquad filter is that an adjustable order r is introduced into the s term of the denominator polynomial, which can be regarded as the combination of the second-order differential term and the fractional second-order low-pass filter [25]. In order to make the fractional second-order low-pass filter meet the standard form of the second-order low-pass filter, that is, the logarithmic amplitude frequency characteristic at the natural frequency is −3 dB [26], design $\zeta_{2-\mathrm{biq}-\theta }=\frac{{\left(2\pi f_{n2-\mathrm{biq}-\theta }\right)}^{1-r}}{\sqrt{2}}$, where $f_{n2-\mathrm{biq}-\theta }$ is the natural frequency and determined according to the position of high frequency noise in the Bode diagram of the control plant in the experimental system. Design $\zeta_{1-\mathrm{biq}-\theta }=\zeta_{\theta }$ in the molecular polynomial, there is a trough in $C_{\mathrm{biq}-\theta }$ and its waveform is the same as the resonance peak of the control plant. Design $f_{n1-\mathrm{biq}-\theta }$ as an adjustable parameter and $f_{n1-\mathrm{biq}-\theta }<f_{n-\theta }$. The filtered control plant is:

(27) $$\begin{array}{lll} P_{\theta -\mathrm{fl}}\left(s\right) & = & P_{\theta }\left(s\right)C_{\mathrm{biq}-\theta }\left(s\right)\\ & = & \frac{K_{f\theta }}{k_{xH}d_{x}H^{2}}\frac{f_{n-\theta }{}^{2}}{f_{n1-\mathrm{biq}-\theta }{}^{2}}\frac{s^{2}+2\zeta_{1-\mathrm{biq}-\theta }2\pi f_{n1-\mathrm{biq}-\theta }s+{\left(2\pi f_{n1-\mathrm{biq}-\theta }\right)}^{2}}{s^{2}+2\zeta_{\theta }2\pi f_{n-\theta }s+{\left(2\pi f_{n-\theta }\right)}^{2}}\cdot \\ & & \frac{{\left(2\pi f_{n2-\mathrm{biq}-\theta }\right)}^{2}}{s^{2}+2\zeta_{2-\mathrm{biq}-\theta }2\pi f_{n2-\mathrm{biq}-\theta }s^{r}+{\left(2\pi f_{n2-\mathrm{biq}-\theta }\right)}^{2}}e^{-\tau s} \end{array},$$

and is a combination of the biquad filter and the fractional second-order low-pass filter. There is a trough and a peak in its amplitude-frequency curve, and the trough is located on the left side of the peak. Its phase-frequency curve first increases and then decreases at $f_{n-\theta }$ and there is no −180° phase crossover point. Therefore, the amplitude of the peak does not need to be limited below 0 dB, and the system gain can be improved.

##### Rz Feedback Controller Design

The Rz feedback controller is a PI controller, $C_{\mathrm{PI}-\theta }=k_{p-\theta }\left(1+\frac{2\pi f_{i-\theta }}{s}\right)$, $k_{p-\theta }$ is the controller gain and $f_{i-\theta }$ is the integration frequency. Under the condition that the order r of $C_{\mathrm{biq}-\theta }$ is given in advance, the system has three adjustable parameters $k_{p-\theta }$, $f_{i-\theta }$ and $f_{n1-\mathrm{biq}-\theta }$, which can satisfy three frequency domain design specifications. When the design specifications are gain crossover frequency $f_{c}$, phase margin $\varphi_{m}$, and gain margin $h_{m}$, the open-loop transmission function $G_{o}\left(s\right)=C_{\mathrm{PI}}\left(s\right)\cdot P_{\theta -\mathrm{fl}}\left(s\right)$ of the system can meet the following constraints [27,28].

1. The phase margin at the gain crossover frequency $f_{c}$ is $\varphi_{m}$.
  (28) $$\begin{array}{c} ∠G_{o}\left(j2\pi f_{c}\right)=∠C_{\mathrm{PI}-\theta }\left(j2\pi f_{c}\right)+∠P_{\theta -\mathrm{fl}}\left(j2\pi f_{c}\right)=-\pi +\varphi_{m} \end{array}.$$
2. The amplitude at the gain crossover frequency $f_{c}$ is 1.
  (29) $$\begin{array}{c} \begin{array}{c} \left|G_{o}\left(j2\pi f_{c}\right)\right|=\left|C_{\mathrm{PI}-\theta }\left(j2\pi f_{c}\right)P_{\theta -\mathrm{fl}}\left(j2\pi f_{c}\right)\right|=1 \end{array} \end{array}.$$
3. The phase at the phase crossover frequency $f_{x}$ is −π.
  (30) $$∠G_{o}\left(j2\pi f_{x}\right)=∠C_{\mathrm{PI}-\theta }\left(j2\pi f_{x}\right)+∠P_{\theta -\mathrm{fl}}\left(j2\pi f_{x}\right)=-\pi .$$
4. The gain margin at the phase crossover frequency $f_{x}$ is $h_{m}$.
  (31) $$\left|G_{o}\left(j2\pi f_{x}\right)\right|=\left|C_{\mathrm{PI}-\theta }\left(j2\pi f_{x}\right)P_{\theta -\mathrm{fl}}\left(j2\pi f_{x}\right)\right|=10^{-\frac{h_{m}}{20}}.$$

From Equation (28), we can obtain,

(32) $$f_{i-\theta }=-f_{c}\tan\left(\varphi_{m}-A_{\theta }\right).$$

where, $\begin{array}{c} A_{\theta }=∠C_{\mathrm{biq}-\theta }\left(j2\pi f_{c}\right)+∠P_{\theta }\left(j2\pi f_{c}\right) \end{array}$.

From Equation (29), we can obtain,

(33) $$k_{p-\theta }=\frac{f_{c}}{B_{\theta }\cdot \sqrt{f_{i-\theta }{}^{2}+f_{c}{}^{2}}}.$$

where, $\begin{array}{c} B_{\theta }=\left|C_{\mathrm{biq}-\theta }\left(j2\pi f_{c}\right)\right|\cdot \left|P_{\theta }\left(j2\pi f_{c}\right)\right| \end{array}$.

From Equation (30), we can obtain,

(34) $$f_{i-\theta }=f_{x}\tan\left(D_{\theta }\right).$$

where, $\begin{array}{c} D_{\theta }=∠C_{\mathrm{biq}-\theta }\left(j2\pi f_{x}\right)+∠P_{\theta }\left(j2\pi f_{x}\right) \end{array}$.

From Equation (31), we can obtain,

(35) $$k_{p-\theta }=\frac{f_{x}10^{-\frac{h_{m}}{20}}}{E_{\theta }\cdot \sqrt{f_{i-\theta }{}^{2}+f_{x}{}^{2}}}.$$

where, $\begin{array}{c} E_{\theta }=\left|C_{\mathrm{biq}-\theta }\left(j2\pi f_{x}\right)\right|\cdot \left|P_{\theta }\left(j2\pi f_{x}\right)\right| \end{array}$.

By substituting Equation (32) into (33), the explicit expression of $k_{p-\theta }$ with respect to $f_{n1-\mathrm{biq}-\theta }$ can be obtained,

(36) $$k_{p-\theta }=\frac{1}{B_{\theta }\cdot \sqrt{\tan^{2}\left(\varphi_{m}-A_{\theta }\right)+1}}.$$

By substituting Equation (32) into (33), the equation of $f_{x}$ and $f_{n1-\mathrm{biq}-\theta }$ can be obtained,

(37) $$f_{x}\tan\left(D_{\theta }\right)=-f_{c}\tan\left(\varphi_{m}-A_{\theta }\right).$$

By substituting Equations (32) and (36) into (35), the other equation of $f_{x}$ and $f_{n1-\mathrm{biq}-\theta }$ can be obtained,

(38) $$\frac{1}{B_{\theta }\cdot \sqrt{\tan^{2}\left(\varphi_{m}-A_{\theta }\right)+1}}=\frac{f_{x}10^{-\frac{h_{m}}{20}}}{E_{\theta }\cdot \sqrt{{\left(f_{c}\tan\left(\varphi_{m}-A_{\theta }\right)\right)}^{2}+f_{x}{}^{2}}}.$$

It is difficult to obtain the analytical solutions of $f_{x}$ and $f_{n1-\mathrm{biq}-\theta }$ as Equations (37) and (38) are complicated. Graphical methods can be used to find the intersection of two equations, and the implicit plots function “ezplot” in MATLAB can be used to draw the curves of the two equations to find the intersection, that is, the solution of the equations. Then, $f_{x}$ and $f_{n1-\mathrm{biq}-\theta }$ can be obtained. $f_{i-\theta }$ can be obtained by substituting $f_{n1-\mathrm{biq}-\theta }$ into Equation (32). $k_{p-\theta }$ can be obtained by substituting $f_{n1-\mathrm{biq}-\theta }$ into Equation (36). To sum up, $k_{p-\theta }$, $f_{i-\theta }$, and $f_{n1-\mathrm{biq}-\theta }$ can be determined.

##### Rz Feed-Forward Controller Design

In order to realize the synchronous movement of the X-direction double motors, the Rz reference input is 0. It is unnecessary to introduce the feed-forward control with input compensation. According to Section 2.1.4 and Section 2.1.5, when the platform moves along the X and Y directions, the Rz will be affected by the disturbance torque, the feed-forward control with disturbance compensation can be introduced to reduce the angle error, and the feed-forward torque model is opposite to the disturbance torque model.

When the platform moves along the Y-direction, the disturbance torque model is shown in Equation (18), in which the feed-forward force $F_{y}$ can be obtained by Equation (3), then the feed-forward controller is designed as,

(39) $$\begin{array}{ll} C_{\mathrm{dff}-ym-\theta }\left(s\right)= & \frac{1}{K_{f\theta }}\frac{m_{x}s^{2}+4c_{xH}s+4k_{xH}}{\left(m_{x}+m_{y}\right)s^{2}+4c_{xH}s+4k_{xH}}m_{y}s^{2}e^{\tau s}\cdot \\ & \left(\begin{array}{c} \frac{m_{x}s^{2}}{m_{x}s^{2}+4c_{xH}s+4k_{xH}}x_{xc}+\frac{J_{Yz}s^{2}}{J_{Yz}s^{2}+c_{yH}d_{yH}{}^{2}s+k_{yH}d_{yH}{}^{2}}x_{F_{y}c}\\ +\frac{c_{yH}d_{yH}{}^{2}s+k_{yH}d_{yH}{}^{2}}{J_{Yz}s^{2}+c_{yH}d_{yH}{}^{2}s+k_{yH}d_{yH}{}^{2}}x_{yc} \end{array}\right) \end{array}$$

The feed-forward current signal to be introduced into the Rz control loop is

(40) $$\begin{array}{ll} i_{\mathrm{dff}-ym-\theta }\left(s\right)= & y_{\mathrm{ref}}C_{\mathrm{dff}-ym-\theta }\left(s\right)=\frac{1}{K_{f\theta }}\frac{m_{x}s^{2}+4c_{xH}s+4k_{xH}}{\left(m_{x}+m_{y}\right)s^{2}+4c_{xH}s+4k_{xH}}m_{y}a_{y}e^{\tau s}\cdot \\ & \left(\begin{array}{c} \frac{m_{x}s^{2}}{m_{x}s^{2}+4c_{xH}s+4k_{xH}}x_{xc}+\frac{J_{Yz}s^{2}}{J_{Yz}s^{2}+c_{yH}d_{yH}{}^{2}s+k_{yH}d_{yH}{}^{2}}x_{F_{y}c}\\ +\frac{c_{yH}d_{yH}{}^{2}s+k_{yH}d_{yH}{}^{2}}{J_{Yz}s^{2}+c_{yH}d_{yH}{}^{2}s+k_{yH}d_{yH}{}^{2}}x_{yc} \end{array}\right) \end{array}$$

When the platform moves along the X-direction, the disturbance torque is shown in Equation (15). In order to compensate for the time-delay in the system, the feed-forward compensation torque can be calculated by the route planned in advance in the XY direction, and the feed-forward controller is designed as,

(41) $$C_{\mathrm{dff}-xm-\theta }\left(s\right)=\frac{1}{K_{f\theta }}\frac{m_{x}m_{y}{}^{2}s^{4}}{\left(m_{y}s^{2}+4c_{yH}s+4k_{yH}\right)\left(m_{x}+m_{y}\right)}e^{\tau s}.$$

The feed-forward current signal to be introduced into the Rz control loop is:

(42) $$\begin{array}{ll} i_{\mathrm{dff}-xm-\theta }\left(s\right) & =x_{\mathrm{ref}}y_{\mathrm{ref}}C_{\mathrm{dff}-xm-\theta }\left(s\right)\\ & =\frac{1}{K_{f\theta }}\frac{m_{x}m_{y}{}^{2}s^{2}}{\left(m_{y}s^{2}+4c_{yH}s+4k_{yH}\right)\left(m_{x}+m_{y}\right)}\left(a_{x}\left(s\right)\cdot e^{\tau s}\right)\cdot \left(y_{\mathrm{ref}}\left(s\right)\cdot e^{\tau s}\right) \end{array}.$$

#### 2.2.2. X and Y Controller Design

It can be seen from Equation (22) that the X control plant is a Resonance-Antiresonance model [29] with time delay and it can be transformed into the following equation,

(43) $$\begin{array}{ll} P_{x}\left(s\right) & =\frac{x_{c}\left(s\right)}{i_{x}\left(s\right)}=\frac{K_{fx}}{\left(m_{x}+m_{y}\right)s^{2}}\frac{m_{y}s^{2}+4c_{yH}s+4k_{yH}}{\frac{m_{x}m_{y}}{m_{x}+m_{y}}s^{2}+4c_{yH}s+4k_{yH}}e^{-\tau s}\\ & =\frac{1}{k_{x}s^{2}}\frac{f_{r-x}{}^{2}}{f_{\mathrm{ar}-x}{}^{2}}\frac{s^{2}+2\zeta_{\mathrm{ar}-x}2\pi f_{\mathrm{ar}-x}s+{\left(2\pi f_{\mathrm{ar}-x}\right)}^{2}}{s^{2}+2\zeta_{r-x}2\pi f_{r-x}s+{\left(2\pi f_{r-x}\right)}^{2}}e^{-\tau s} \end{array},$$

where, $f_{\mathrm{ar}-x}=\frac{1}{\pi }\sqrt{\frac{k_{yH}}{m_{y}}}$, $\zeta_{\mathrm{ar}-x}=\frac{c_{yH}}{\sqrt{m_{y}k_{yH}}}$, $\;f_{r-x}=\frac{1}{\pi }\sqrt{\frac{k_{yH}\left(m_{x}+m_{y}\right)}{m_{x}m_{y}}}$, $\;\zeta_{\mathrm{ar}-y}=c_{yH}\sqrt{\frac{\left(m_{x}+m_{y}\right)}{k_{yH}m_{x}m_{y}}}$, $k_{x}=\frac{m_{x}+m_{y}}{K_{fx}}$. The biquad filter is introduced in the control loop to match and cancel the resonance and antiresonance term, $C_{\mathrm{biq}-x}=\frac{f_{n2-\mathrm{biq}-x}{}^{2}}{f_{n1-\mathrm{biq}-x}{}^{2}}\frac{s^{2}+2\zeta_{1-\mathrm{biq}-x}2\pi f_{n1-\mathrm{biq}-x}s+{\left(2\pi f_{n1-\mathrm{biq}-x}\right)}^{2}}{s^{2}+2\zeta_{2-\mathrm{biq}-x}2\pi f_{n2-\mathrm{biq}-x}s+{\left(2\pi f_{n2-\mathrm{biq}-x}\right)}^{2}}$ and design $f_{n1-\mathrm{biq}-x}=f_{r-x}$, $\zeta_{1-\mathrm{biq}-x}=\zeta_{r-x}$, $f_{n2-\mathrm{biq}-x}=f_{\mathrm{ar}-x}$ and $\zeta_{2-\mathrm{biq}-x}=\zeta_{\mathrm{ar}-x}$. The second-order low-pass filter is introduced in the loop to suppress the high-frequency noise, $C_{\mathrm{lp}2-x}=\frac{{\left(2\pi f_{n-\mathrm{lp}2-x}\right)}^{2}}{s^{2}+2\zeta_{\mathrm{lp}2-x}2\pi f_{n-\mathrm{lp}2-x}s+{\left(2\pi f_{n-\mathrm{lp}2-x}\right)}^{2}}$, $\zeta_{\mathrm{lp}2-x}$ = 0.707 and $f_{n-\mathrm{lp}2-x}$ is determined according to the position of high frequency noise in the experimental system. The filtered control plant is

(44) $$P_{x-\mathrm{fl}}\left(s\right)=\frac{1}{k_{x}s^{2}}\frac{{\left(2\pi f_{n-\mathrm{lp}2-x}\right)}^{2}}{s^{2}+2\zeta_{\mathrm{lp}2-x}2\pi f_{n-\mathrm{lp}2-x}s+{\left(2\pi f_{n-\mathrm{lp}2-x}\right)}^{2}}e^{-\tau s}.$$

The X feedback controller is the PID controller, $C_{\mathrm{PID}-x}=k_{p-x}\left(1+\frac{2\pi f_{i-x}}{s}+\frac{s}{2\pi f_{d-x}}\right)$. $k_{p-x}$ is the controller gain, $f_{i-x}$ is the integration frequency, and $f_{d-x}$ is the differential frequency. Since there are three control parameters, the open-loop transfer function of the system can meet three design specifications. When the design specifications are gain crossover frequency $f_{c}$, phase margin $\varphi_{m}$, and gain margin $h_{m}$, the calculation method of control parameters in X-direction is shown in the Appendix A.

The system implements the point-to-point motion in X-direction and the reference position $x_{\mathrm{ref}}$ is a fourth-order planning path. The feed-forward control with input compensation is introduced to improve the position tracking accuracy, and the feed-forward controller is the inverse model of the X control plant,

(45) $$C_{\mathrm{ff}-x}\left(s\right)=\frac{\frac{m_{x}m_{y}}{m_{x}+m_{y}}s^{2}+4c_{yH}s+4k_{yH}}{m_{y}s^{2}+4c_{yH}s+4k_{yH}}\frac{m_{x}+m_{y}}{K_{fx}}s^{2}e^{\tau s}.$$

The feed-forward current signal of X loop can be calculated by using the acceleration signal planned in advance [30].

(46) $$i_{\mathrm{ff}-x}\left(s\right)=x_{\mathrm{ref}}C_{\mathrm{ff}-x}\left(s\right)=\frac{\frac{m_{x}m_{y}}{m_{x}+m_{y}}s^{2}+4c_{yH}s+4k_{yH}}{m_{y}s^{2}+4c_{yH}s+4k_{yH}}\frac{m_{x}+m_{y}}{K_{fx}}a_{\mathrm{ref}}e^{\tau s}.$$

It can be seen from Equation (3) that the Y control plant $P_{y}$ is the same as $P_{x}$ in form and it can be transformed into the following equation,

(47) $$\begin{array}{ll} P_{y}\left(s\right) & =\frac{y\left(s\right)}{i_{y}\left(s\right)}=\frac{K_{fy}}{m_{y}s^{2}}\frac{\left(m_{x}+m_{y}\right)s^{2}+4c_{xH}s+4k_{xH}}{m_{x}s^{2}+4c_{xH}s+4k_{xH}}e^{-\tau s}\\ & =\frac{1}{k_{y}s^{2}}\frac{f_{r-y}{}^{2}}{f_{\mathrm{ar}-y}{}^{2}}\frac{s^{2}+2\zeta_{\mathrm{ar}-y}2\pi f_{\mathrm{ar}-y}s+{\left(2\pi f_{\mathrm{ar}-y}\right)}^{2}}{s^{2}+2\zeta_{r-y}2\pi f_{r-y}s+{\left(2\pi f_{r-y}\right)}^{2}}e^{-\tau s} \end{array}.$$

where, $f_{\mathrm{ar}-y}=\frac{1}{\pi }\sqrt{\frac{k_{xH}}{m_{x}+m_{y}}}$, $\zeta_{\mathrm{ar}-y}=\frac{c_{xH}}{\sqrt{\left(m_{x}+m_{y}\right)k_{xH}}}$, $f_{r-y}=\frac{1}{\pi }\sqrt{\frac{k_{xH}}{m_{x}}}$, $\zeta_{\mathrm{ar}-y}=\frac{c_{xH}}{\sqrt{m_{x}k_{xH}}}$, $k_{y}=\frac{m_{y}}{K_{fy}}$. Then the biquad filter and second-order low-pass filter are used for filtering, and the Y feedback controller is the PID controller. Under the same design specifications, the feedback control parameters can be determined according to the design method in the Appendix A. The feed-forward control with input compensation is introduced into the Y loop, and the feed-forward controller is:

(48) $$C_{\mathrm{ff}-y}\left(s\right)=\frac{m_{x}s^{2}+4c_{xH}s+4k_{xH}}{\left(m_{x}+m_{y}\right)s^{2}+4c_{xH}s+4k_{xH}}\frac{m_{y}}{K_{fy}}s^{2}e^{\tau s}.$$

The feed-forward current signal can be calculated by using the acceleration signal planned in advance.

(49) $$i_{\mathrm{ff}-y}\left(s\right)=y_{\mathrm{ref}}C_{\mathrm{ff}-y}\left(s\right)=\frac{m_{x}s^{2}+4c_{xH}s+4k_{xH}}{\left(m_{x}+m_{y}\right)s^{2}+4c_{xH}s+4k_{xH}}\frac{m_{y}}{K_{fy}}a_{\mathrm{ref}}e^{\tau s}.$$

## 3. Results

### 3.1. Simulation Illustration

In this section, Sim-Mechanics is used to build the mechanical model of an H-type motion platform to verify the proposed synchronization control method. Sim-Mechanics is a mechanical simulation module in MATLAB Simulink. It can establish the rigid body model of the mechanical system to realize electromechanical co-simulation with the control module in Simulink. The simulation runs in a Simulink environment with a fixed step size. In order to show the advantages of the proposed method in disturbance rejection performance, the control effect of the proposed mismatched filtering method is compared with the matched filtering method in the literature. The control effect of the proposed fractional order biquad filter method is compared with that of the integer order biquad filter method to show the advantage of the fractional order filter.

**Figure 6:**
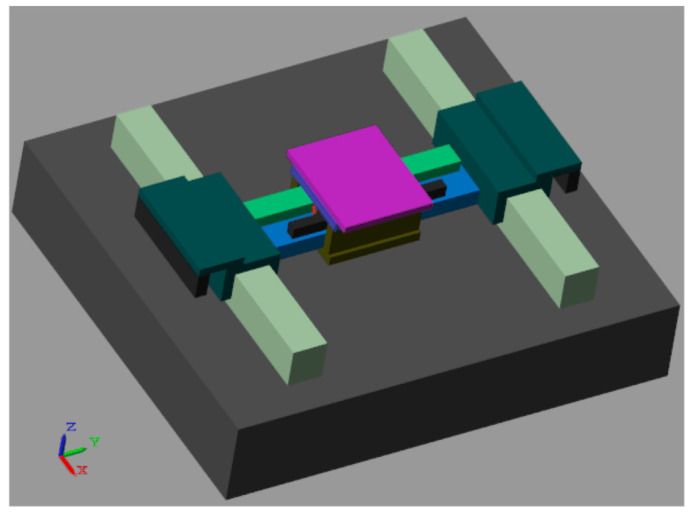
The Sim-Mechanics mechanical model in the simulation.

As shown in Figure 6, the mechanical model is constructed according to the experimental parameters in Table 1, and the control system is built in Simulink. The control period is 5.0 × 10^−4^ s.

#### 3.1.1. Simulation of Feedback Control Effect

**Figure 7:**
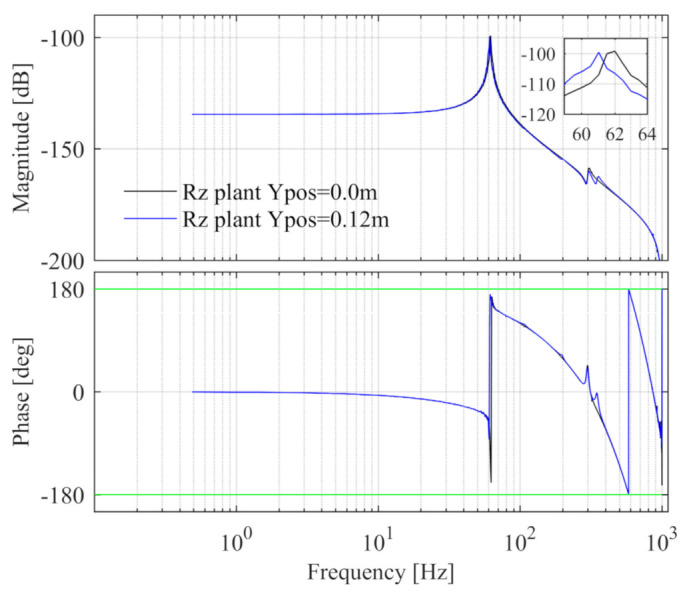
The Bode diagram of the Rz control plant in the simulation.

The Bode diagram of the Rz control plant is shown in Figure 7; there is a resonance peak in the amplitude-frequency characteristic curve, and the phase decreases rapidly from 0° to −180° at the resonance frequency. When the Y component is located in the middle and one side of the beam, the plants are shown as the black and blue line, respectively. The frequency of the resonance peak of the blue line is 1 Hz lower than the black line, because when the Y component deviates from the middle position, the rotational inertia of the entire moving component increases.

**Figure 8:**
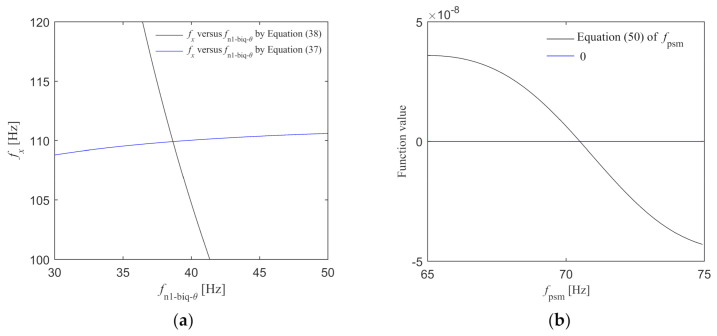
$f_{x}$, $f_{n1-\mathrm{biq}-\theta }$, and $f_{\mathrm{psm}}$ are determined by graphical method. (**a**) $f_{x}$ versus $f_{n1-\mathrm{biq}-\theta }$; (**b**) The solution of the equation of $f_{\mathrm{psm}}$.

The design specifications are $f_{c}$ = 10 Hz, $\varphi_{m}$ = 82°, and $h_{m}$ = 10 dB. Design $f_{n2-\mathrm{biq}-\theta }$ = 300 Hz. The control parameters are calculated based on the control plant when the Y component is in the middle position. With the order r = 1, the parameters $k_{p-\theta }$, $f_{i-\theta }$, and $f_{n1-\mathrm{biq}-\theta }$ are calculated according to the method described in Rz Control Plant. As shown in Figure 8a, the values of $f_{x}$ and $f_{n1-\mathrm{biq}-\theta }$ can be obtained from the the intersection point of Equations (37) and (38), $f_{x}=$109.935 Hz and $f_{n1-\mathrm{biq}-\theta }=$38.659 Hz. $f_{n1-\mathrm{biq}-\theta }$ is substituted into Equation (32) to get $f_{i-\theta }=$808.683 Hz. $f_{n1-\mathrm{biq}-\theta }$ is substituted into Equation (36) to get $k_{p-\theta }=$75545.3. The parameters of Rz feedback controller are determined. On this basis, in order to obtain the maximum amplitude of the process sensitive transfer function $G_{\mathrm{ps}-\theta }=P_{\theta }/\left(1+G_{o}\right)$, the frequency $f_{\mathrm{psm}}$ at the peak point of $G_{\mathrm{ps}-\theta }$ should be calculated. Since the derivative of $\left|G_{\mathrm{ps}-\theta }\right|$ at $f_{\mathrm{psm}}$ is zero, the equation about $f_{\mathrm{psm}}$ can be obtained as Equation (50). As shown in Figure 8b, the value of $f_{\mathrm{psm}}$ can be obtained from the intersection point of Equation (50) and 0 axis, $f_{\mathrm{psm}}$ = 70.504 Hz. Then the logarithmic amplitude at the peak point can be obtained as $G_{\mathrm{psm}-\theta }$ = –121.43 dB.

(50) $${\left.\frac{d\left|G_{\mathrm{ps}-\theta }\right|}{df}\right|}_{f=f_{\mathrm{psm}}}=0.$$

**Figure 9:**
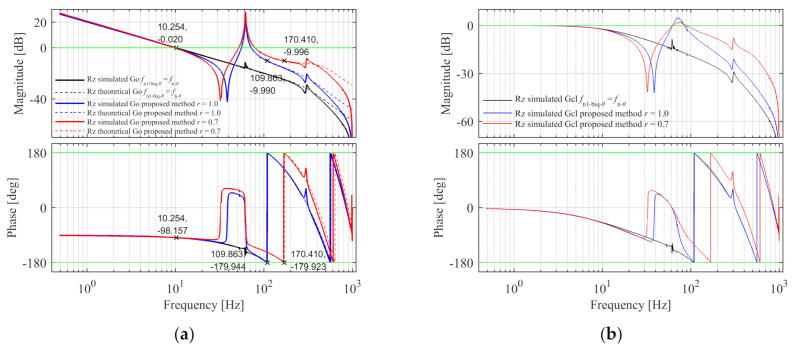

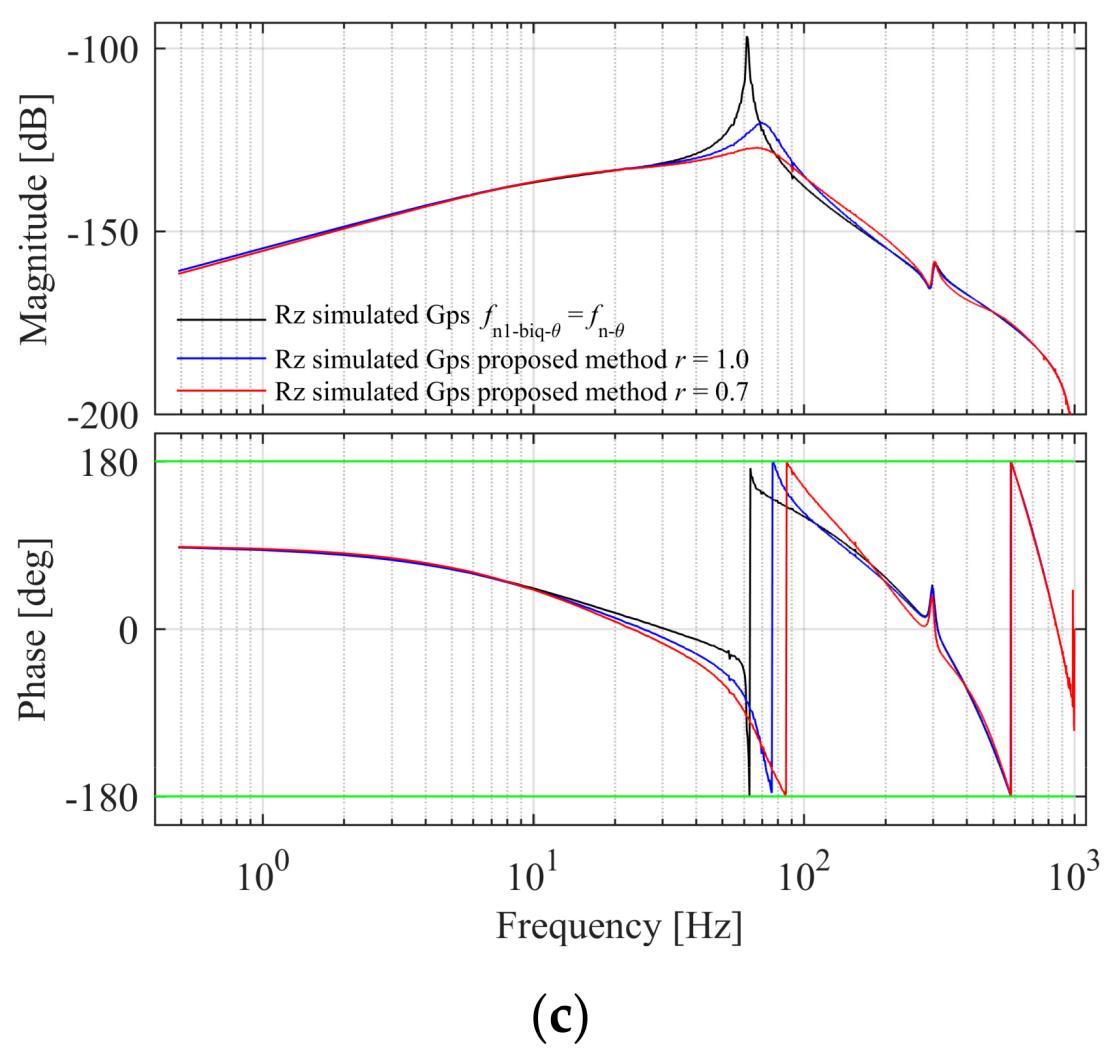
Rz open-loop, close-loop, and process sensitive Bode diagram in the simulation. (**a**) The open-loop Bode diagram; (**b**) The close-loop Bode diagram; (**c**) The process sensitive Bode diagram.

The control parameters and the peak value of the process sensitive transfer function are calculated under different orders, and the results are shown in Table 2. For comparison, when the order r = 1, $f_{n1-\mathrm{biq}-\theta }$ is designed to match the natural frequency $f_{n-\theta }$ of the control plant based on the method of the literature, the process sensitive transfer function gets the peak value at $f_{n-\theta }$, and the data is in the last row of the table. It can be seen that the system has the best disturbance rejection performance when the order is 0.7. Compared with the fixed integer order biquad filter, the introduction of fractional order provides another degree of freedom for parameter tuning, and the order can be selected to achieve better disturbance rejection performance. Figure 9 are the Bode diagrams of the open-loop, close-loop, and process sensitive transfer functions of the theoretical continuous model with r = 1.0 and r = 0.7, and r = 1.0, $f_{n1-\mathrm{biq}-\theta }=f_{n-\theta }$, the solid line is the Sim-Mechanics model and the dashed line is the theoretical model. There are errors between the simulated model and the theoretical open-loop Bode diagrams. The specification errors of the proposed method with r = 1.0 and r = 0.7 are shown in Table 3 and the design specifications of Rz simulated model are satisfied. The reasons for the error are analyzed as follows: (1) The discretization of the continuous model results in errors. In the theoretical model, the controller parameters are calculated and the open-loop Bode diagram is drawn based on the continuous dynamic model and feedback controller model. In the simulated model, the control plant is the discretized model after zero-order-hold discretization, the feedback controller is the discretized model after Tustin discretization, and the fractional term $s^{r}$ is discretized by the impulse response invariant discretization method. There are errors between the Bode diagrams of continuous and discretized models. (2) The existence of unmodeled factors in the control plant will also cause errors. In the mechanical model of simulation, the force acting point of the Y motor does not coincide with its centroid along the X and Z directions, which leads to eccentric driving and causes antiresonance and resonance peaks in the high frequency part. This factor is not considered in the Y-direction theoretical dynamic model, which leads to the deviation of the high frequency part of the open-loop Bode diagram between the simulated and theoretical model. In the X and Rz directions, the force acting point of the X motor does not coincide with the centroid of X moving component along Y and Z directions, which leads to the error of the simulated and theoretical model.

In the control method of the literature, $f_{n1-\mathrm{biq}-\theta }=f_{n-\theta }$, the trough in the filter and the peak in the plant match and cancel each other. As shown in Figure 9a, the amplitude-frequency and phase-frequency curves of the open-loop transfer function are smooth, and the system has enough gain margin and phase margin to ensure stability. As shown in Figure 9c, there is a peak in the process sensitive transfer function at the peak frequency of the plant. The reason is that $G_{\mathrm{ps}-\theta }$ is the combination of the plant $P_{\theta }$ and the sensitivity transfer function $G_{s-\theta }=1/\left(1+G_{o}\right)$. Since there is a peak in $P_{\theta }$, and $G_{o}$ is a smooth curve and $1+G_{o}$ is also a smooth curve, there will be a peak in the combination of $P_{\theta }$ and $G_{s-\theta }$, and the attenuation ability of the system to the disturbance input in front of the control plant is weak, so the system has poor disturbance rejection performance.

In the proposed Rz control method, as shown in Figure 9a, the open-loop amplitude frequency curve is not smooth due to the existence of trough and peak. The troughs are on the left side of the peak and there are three 0 dB gain crossover points, but the phase near the natural frequency of the plant is greatly improved. Under the design specifications, the phase margins at the three gain crossover points are sufficient, and the gain margin at the phase crossover point is 10 dB, so the system stability can be guaranteed. As shown in Figure 9c, the peak value of the process sensitive transfer function in the proposed method is obviously smaller than the method of the literature. The reason is that there is a peak at the natural frequency of the plant in $G_{o}$, and its logarithmic amplitude is greater than 0 dB, which can attenuate the peak value of $P_{\theta }$ in $G_{\mathrm{ps}-\theta }$, so the attenuation ability of the system to the disturbance input in front of the control plant is improved.

**Figure 10:**
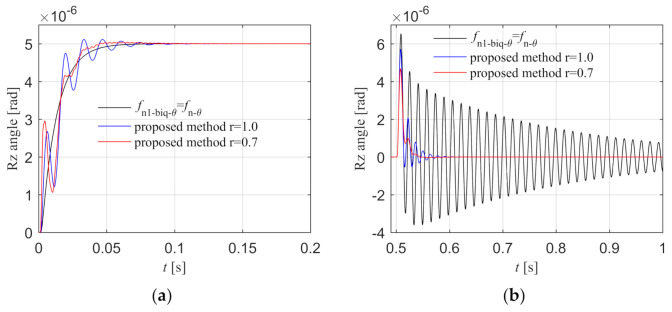
Rz step input and disturbance response in the simulation. (**a**) Step input response; (**b**) Step disturbance response.

The three groups of control parameters are applied to the Rz loop in the simulation model, and the step input response and step disturbance response diagrams are shown in Figure 10a,b. From the step input response curve, it can be seen that the response curve of the trough and peak cancellation filtering method of the literature is relatively smooth and without overshoot. There are fluctuations and overshoots in the proposed method, and the fluctuations and overshoots of the fractional order biquad filter are smaller than those of the integer order filter. From the step disturbance response curve, it can be seen that the peak value of the disturbance is the largest and the oscillation time is longer in the method of the literature. The proposed control method can quickly attenuate the fluctuation caused by the disturbance. Compared with integer order, the peak value of fractional order disturbance response is smaller and the fluctuation is less. The proposed fractional order filtering method has better disturbance rejection performance. To sum up, the step response effect of the method in the literature is better than that of the proposed method, but its disturbance rejection performance is worse than the proposed method. Since the reference input in Rz loop is 0 and remains unchanged, the system requires higher disturbance rejection performance, so the proposed method is more suitable for the synchronous control of the dual motors.

**Figure 11:**
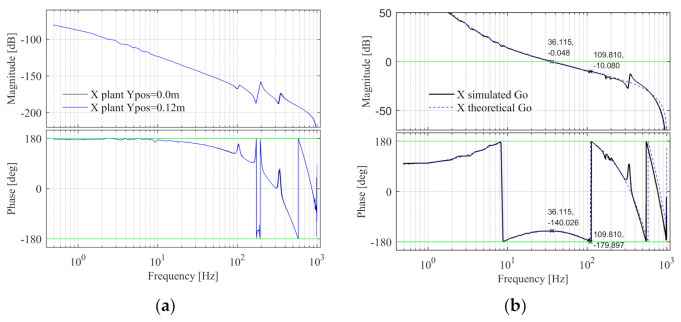
The X control plant and open-loop Bode diagram in the simulation. (**a**) The control plant Bode diagram; (**b**) The open-loop Bode diagram.

The Bode diagram of the X control plant is shown in Figure 11a, when the Y component is located in the middle and one side of the beam, the plants are shown as the black and blue lines respectively, which are basically the same, since the change of the Y component position will not affect the mass of the entire component and the X control plant will not change. The design specifications are $f_{c}$ = 36 Hz, $\varphi_{m}$ = 40°, and $h_{m}$ = 10 dB. Design $f_{n-\mathrm{lp}2-x}$ = 600 Hz, the feedback controller parameters are calculated according to the method described in Appendix A. Calculate $k_{p-x}$ from Equation (A12), $k_{p-x}$ = 7.296 × 10^6^. The intersection point of Equation (A13) and axis 0 is found by graphic method and the phase crossover frequency is obtained, $f_{x}$ = 110.051 Hz. Then calculate $f_{d-x}$ from Equation (A14), $f_{d-x}$ = 14.663 Hz. Then $f_{d-x}$ is substituted into Equation (A18) to get $f_{i-x}$ = 3.991 Hz. The X open-loop Bode diagram is shown in Figure 11b, the black line is the Sim-Mechanics model, and the blue dashed line is the theoretical model. The specification errors between the X simulated and theoretical open-loop Bode diagrams are shown in Table 4 and the design specifications of the X simulated model are satisfied.

**Figure 12:**
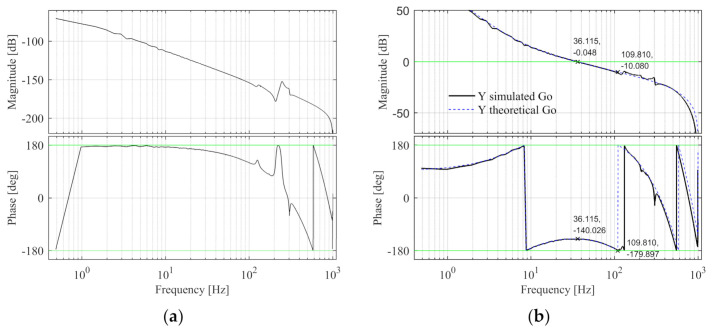
The Y control plant and open-loop Bode diagram in the simulation. (**a**) The control plant Bode diagram; (**b**) The open-loop Bode diagram.

In the Sim-Mechanics model, the Bode diagram of the Y control plant is shown in Figure 12a. The design specifications are same with X-direction, design $f_{n-\mathrm{lp}2-y}$ = 600 Hz and the PID controller parameters are calculated, $k_{p-y}$ = 2.187 × 10^6^, $f_{i-y}$ = 3.991 Hz, and $f_{d-y}$ = 14.663 Hz. The open-loop Bode diagram of the system is shown in Figure 12b, the black line is the Sim-Mechanics model, and the blue dashed line is the theoretical model. The specification errors between the Y simulated and theoretical open-loop Bode diagrams are shown in Table 5 and the design specifications of Y simulated model are satisfied.

#### 3.1.2. Simulation of Feed-Forward Control Effect

**Figure 13:**
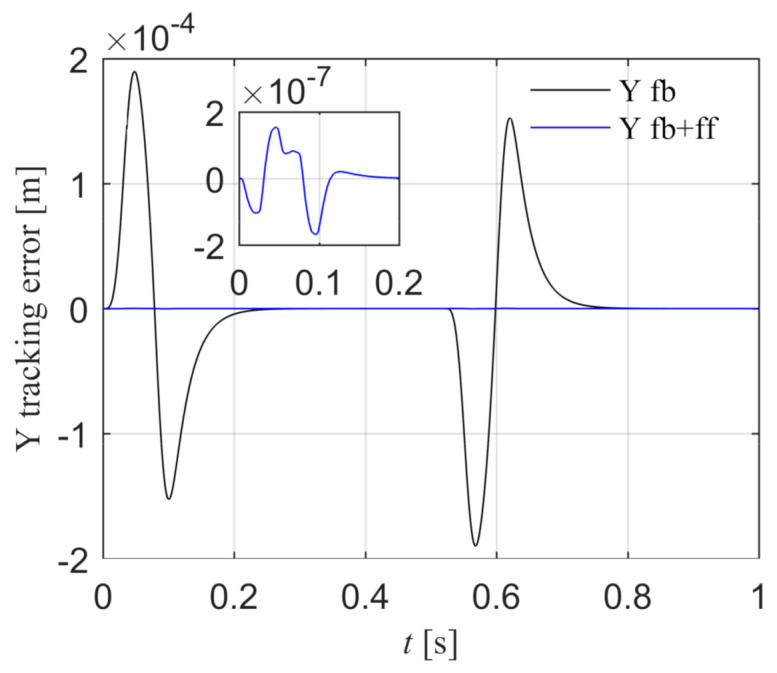
Comparison of Y position errors with and without feed-forward control in the simulation.

The Y reference position $y_{\mathrm{ref}}$ is a fourth-order point-to-point motion path, the peak values of position, velocity, acceleration, jerk, and spasm are 0.13 m, 0.25 m/s, 5.0 m/s^2^, 1000.0 m/s^3^, and 10,000.0 m/s^4^. The Y tracking error is shown in Figure 13. The black line is the position tracking error under the feedback control, and the peak value is 1.898 × 10^−4^ m. The blue line is the position tracking error after introducing the feed-forward control according to Equation (49), and the peak error is 1.536 × 10^−7^ m. The tracking accuracy is greatly improved, which indicates that the Y feed-forward control model is correct.

**Figure 14:**
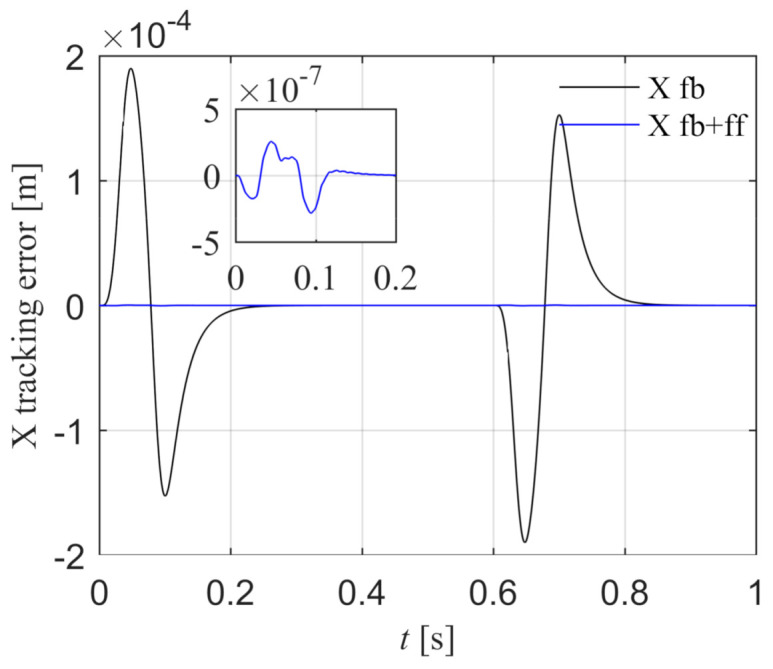
Comparison of X position errors with and without feed-forward control in the simulation.

The X reference position $x_{\mathrm{ref}}$ is a fourth-order point-to-point motion path, the peak values of position, velocity, acceleration, jerk, and spasm are 0.15 m, 0.25 m/s, 5.0 m/s^2^, 1000.0 m/s^3^, and 10000.0 m/s^4^. The X tracking error is shown in Figure 14. The black line is the position tracking error under the feedback control, and the peak value is 1.899 × 10^−4^ m. The blue line is the position tracking error after introducing the feed-forward control according to Equation (46), and the peak error is 2.838 × 10^−7^ m. The tracking accuracy is greatly improved, which indicates that the X feed-forward control model is correct.

In the Rz loop, the proposed control method with r = 0.7 in the fractional order filter is applied. The platform executes point-to-point motion in X and Y directions, and the Rz feed-forward compensation method of disturbance torque is verified.

1. X component is stationary and Y component moves.

**Figure 15:**
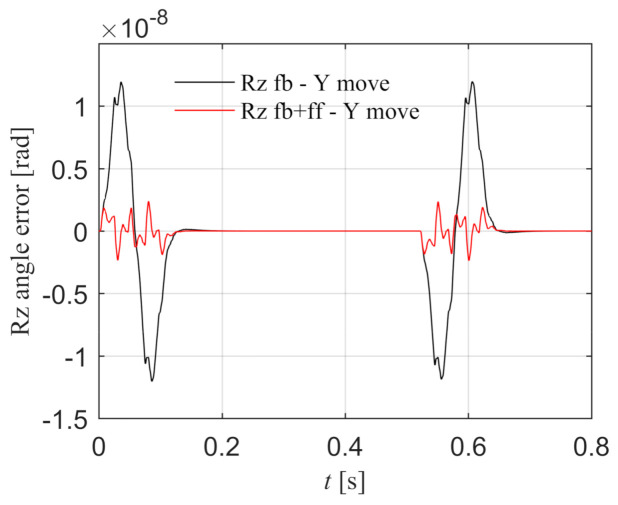
When X component is stationary and Y component moves, the Rz angle error with and without feed-forward control in the simulation.

As shown in Figure 15, the black line is the Rz angle error under the feedback control, which shows that the Y component motion will cause the synchronization error, and the peak error is 1.195 × 10^−8^ rad. The red line is the Rz angle error with torque feed-forward control according to Equation (40), which can reduce the synchronization error caused by the disturbance torque, and the peak error is 2.381 × 10^−9^ rad. The synchronization error is significantly reduced, which proves that the Rz torque feed-forward is effective when Y component moves.

2. X component moves and Y component is stationary.

**Figure 16:**
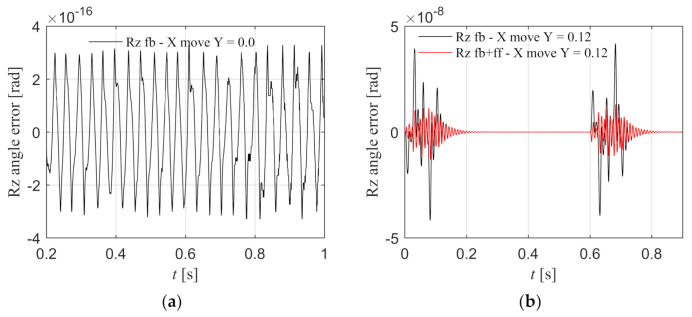
When X component moves, the Rz angle error with and without feed-forward control in the simulation. (**a**) Y component is stationary in the middle position of the beam; (**b**) Y component is stationary in the one side position of the beam.

The Y component is stationary in the middle position of the beam, and the Rz angle error under the feedback control is shown as the black line in Figure 16a, and the angle error is almost zero. It can be seen that when Y component is in the middle position, the X component motion will not cause the angle error. When the Y component is located at one side position of the beam, the Rz angle error under the feedback control is shown as the black line in Figure 16b, and the peak error is 4.180 × 10^−8^ rad. It can be seen that when the Y component is not located in the middle position, the X component motion will cause the angle error, which is consistent with the disturbance torque model mentioned above. The red line is the Rz angle error with torque feed-forward control according to Equation (42), and the peak error is 1.140 × 10^−8^ rad. The synchronization error is significantly reduced, which proves that the Rz torque feed-forward is effective when the X component moves.

### 3.2. Experimental Verification and Analisys of Results

#### 3.2.1. Experimental Setup

In this section, the proposed synchronization control method is verified on the H-type air floating motion platform. The experimental setup is shown in Figure 1. The controller is equipped with a real-time network card supporting EtherCAT communication. The controller communicates with the three motor drivers through EtherCAT bus to drive the motors, and the position signal is measured by the grating ruler and fed back to the controller to realize the closed-loop control. The RELM grating ruler is used in the platform and the overall accuracy is achieved ±1 um. The grating pitch is 20 um and the resolution after subdivision is 4.883 nm. The system software includes a monitoring software (Twincat 3.0 Scope View) and a real-time control software (Twincat 3.0 eXtended Automation Engineering). The control algorithm is implemented by using C/C++ code-based modules and the sampling period is 5.0 × 10^−4^ s.

#### 3.2.2. Experimental Verification of Feedback Control Effect

**Figure 17:**
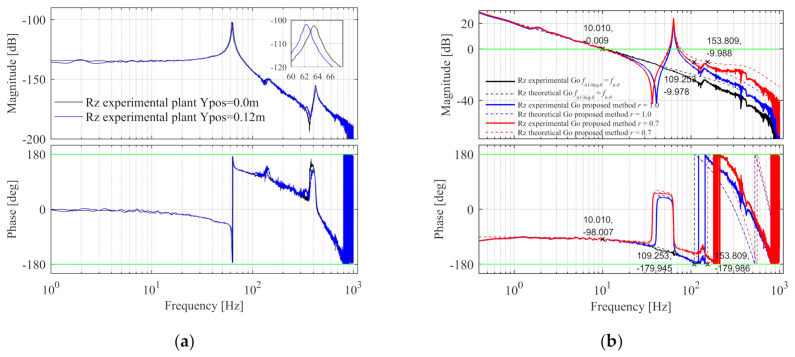

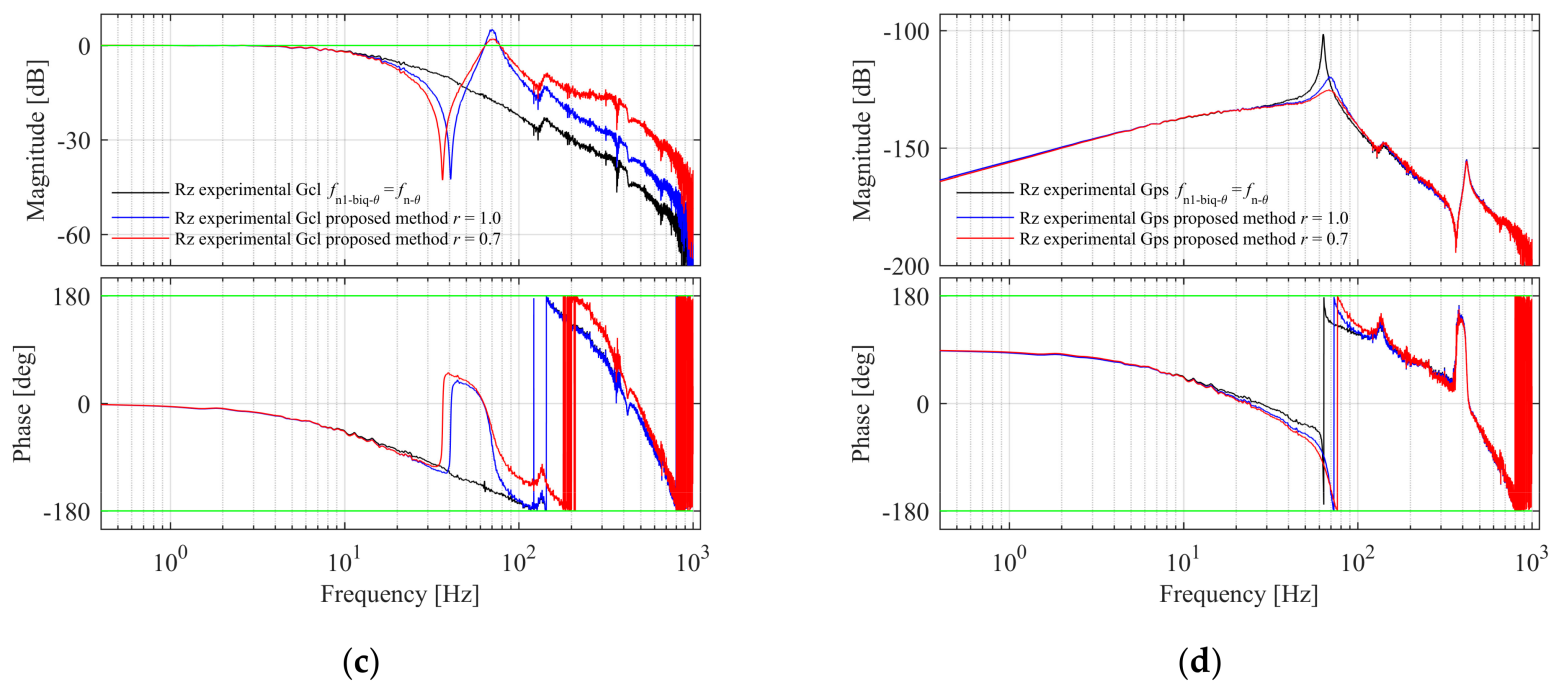
The Rz control plant, open-loop, close-loop, and process sensitive Bode diagram in the experiment. (**a**) The control plant Bode diagram; (**b**) The open-loop Bode diagram; (**c**) The close-loop Bode diagram; (**d**) The process sensitive Bode diagram.

In the experimental system, the Bode diagram of the Rz control plant is shown in Figure 17a, when the Y component position is 0.0 and 0.12 m, the plants are shown as the black and blue line, respectively. The frequency of the resonance peak of the blue line is 1 Hz lower than the black line, because when the Y component deviates from the middle position, the rotational inertia of the entire moving component increases. The Rz feedback control parameters are determined by the method of the simulation model to satisfy the same design specification. Figure 17b–d are the Bode diagrams of the open-loop, close-loop, and process sensitive transfer functions with r = 1.0 and r = 0.7, and r = 1.0, $f_{n1-\mathrm{biq}-\theta }=f_{n-\theta }$. In Figure 17b. The solid line is the experimental model and the dashed line is the theoretical model. The specification errors in proposed method with r = 1.0 and r = 0.7 are shown in Table 6. It can be seen that the dashed line satisfies the design specifications. Due to the discrete implementation of the continuous transfer function and other unmodeled factors, the gain margin of the experimental model deviates from the theoretical model in the high frequency part, but the stability of the system is not affected.

**Figure 18:**
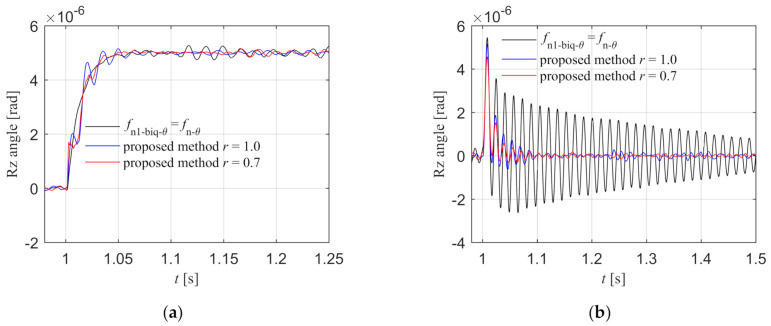
Rz step input and disturbance response in the experiment. (**a**) Step input response; (**b**) Step disturbance response.

The three groups of control parameters are applied to the Rz loop of the experimental system, and the step input response and step disturbance response diagrams are shown in Figure 18a,b. From the step input response curve, it can be seen that the response curve of the trough and peak cancellation filtering method of the literature is relatively smooth and without overshoot. There are fluctuations and overshoots in the proposed method, and the fluctuations and overshoots of the fractional order biquad filter are smaller than those of the integer order filter. From the step disturbance response curve, it can be seen that the peak value of the disturbance response is the largest and the oscillation time is the longest in the method of the literature. The proposed control method can quickly attenuate the fluctuation caused by the disturbance. Compared with integer order, the peak value of disturbance response is smaller and the fluctuation is less in the proposed fractional order filtering method. The proposed fractional order filtering method has better disturbance rejection performance. To sum up, the step response effect of the method in the literature is better than that of the proposed method, but its disturbance rejection performance is worse than the proposed method. Since the Rz reference input is 0 and remains unchanged, the system requires higher disturbance rejection performance, so the proposed method is more suitable for the synchronous control of the dual motors.

**Figure 19:**
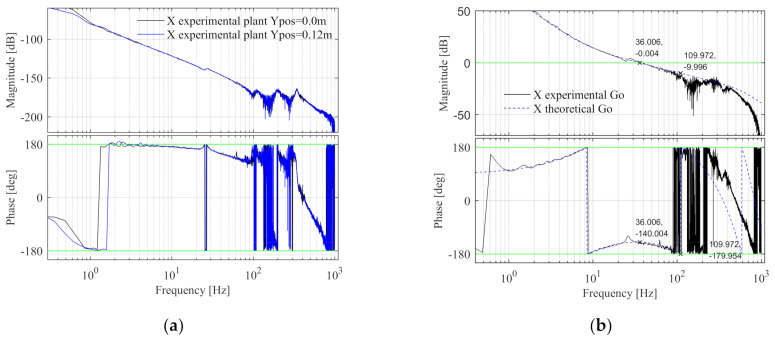
The X control plant and open-loop Bode diagram in the experiment. (**a**) The control plant Bode diagram; (**b**) The open-loop Bode diagram.

The Bode diagram of the X control plant is shown in Figure 19a, when the Y component position is 0.0 and 0.12 m, the plants are shown as the black and blue lines respectively, which are basically the same. It can be seen that the change of Y component position will not affect the X control plant. The design specifications and control parameters of the X simulation model are adopted here. The open-loop Bode diagram of the system is shown in Figure 19b. The black line is the experimental model and the blue line is the theoretical model. The specification errors are shown in Table 7. It can be seen that the design specifications of X experimental model are satisfied.

**Figure 20:**
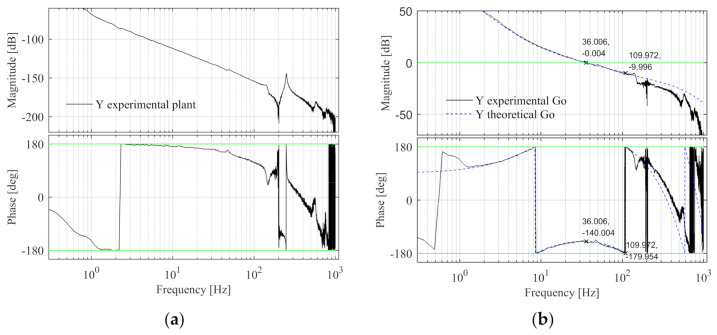
The Y control plant and open-loop Bode diagram in the experiment. (**a**) The control plant Bode diagram; (**b**) The open-loop Bode diagram.

The Bode diagram of the Y control plant is shown in Figure 20a. The design specifications and control parameters of the Y simulation model are adopted here. The Y open-loop Bode diagram is shown in Figure 20b. The black line is the experimental model and the blue line is the theoretical model. The specification errors are shown in Table 8. It can be seen that the design specifications of Y experimental model are satisfied.

#### 3.2.3. Experimental Verification of Feed-Forward Control Effect

**Figure 21:**
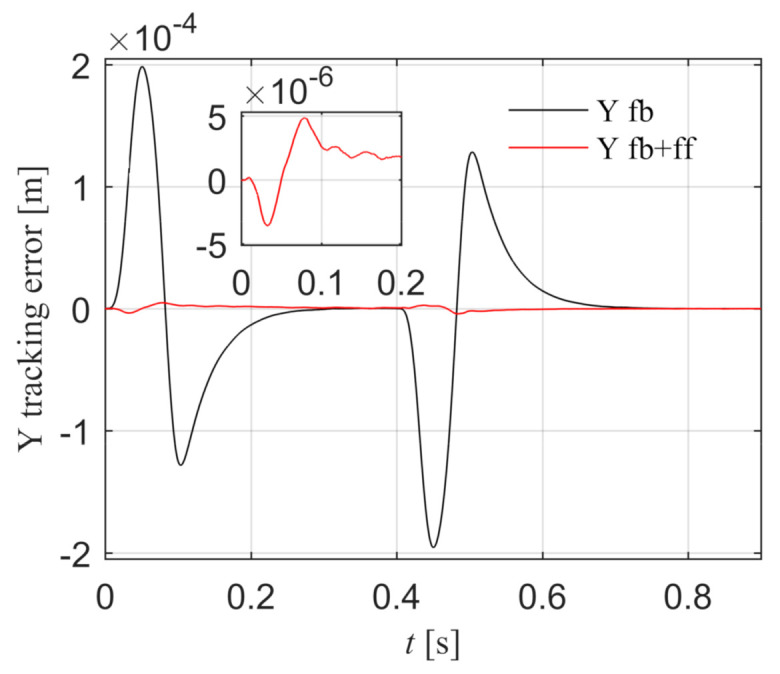
Comparison of Y position errors with and without feed-forward control in the experiment.

In point-to-point motion experiments, the X and Y reference paths are same with the simulation model. The X tracking error is shown in Figure 21. The black line is the position tracking error under the feedback control, and the peak value is 1.985 × 10^−4^ m. The blue line is the position tracking error after introducing the feed-forward control according to Equation (49), and the peak error is 4.790 × 10^−6^ m. The tracking accuracy is greatly improved, which indicates that the Y feed-forward control model is correct.

**Figure 22:**
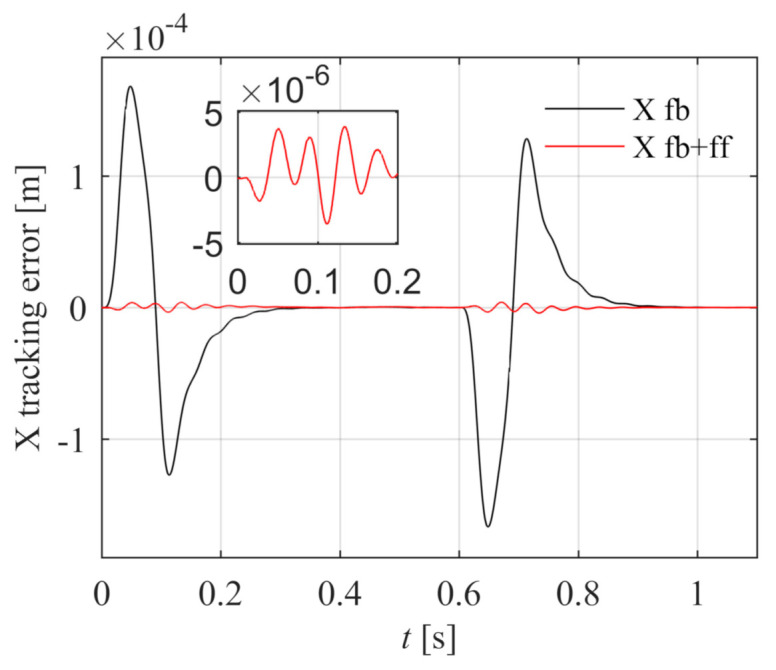
Comparison of X position errors with and without feed-forward control in the experiment.

The X tracking error is shown in Figure 22. The black line is the position tracking error under the feedback control, and the peak value is 1.669 × 10^−4^ m. The blue line is the position tracking error after introducing the feed-forward control according to Equation (46), and the peak error is 3.553 × 10^−6^ m. The tracking accuracy is greatly improved, which indicates that the X feed-forward control model is correct.

In the Rz loop, the proposed control method with r = 0.7 in the fractional order filter is applied. The platform executes point-to-point motion in X and Y directions, and the Rz feed-forward compensation method of disturbance torque is verified.

1. X component is stationary and Y component moves.

**Figure 23:**
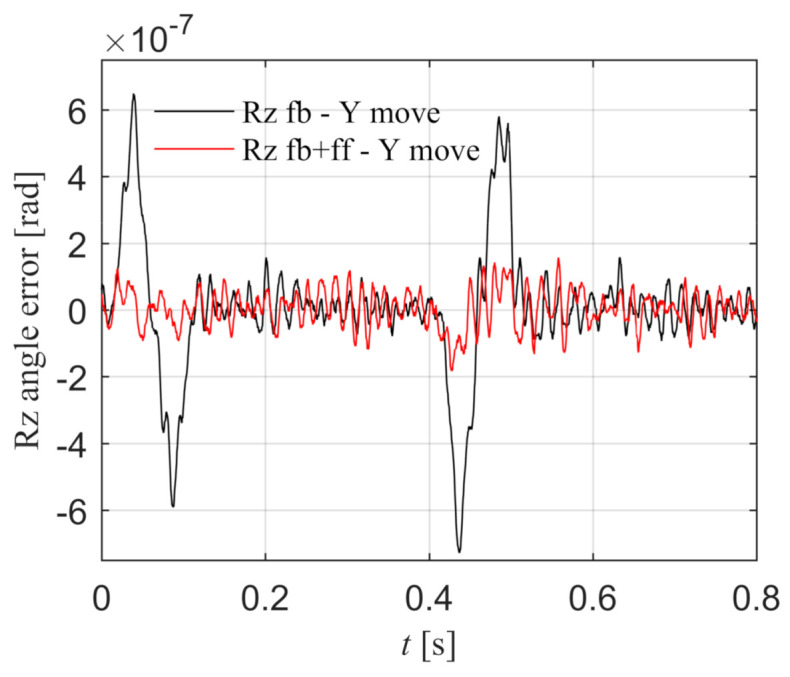
When X component is stationary and Y component moves, the Rz angle error with and without feed-forward control in the experiment.

As shown in Figure 23, the black line is the Rz angle error under the feedback control, which shows that the movement of the Y component will cause the synchronization error, and the peak error is 7.285 × 10^−7^ rad. The red line is the Rz angle error with torque feed-forward control according to Equation (40), and the peak error is 1.821 × 10^−7^ rad. The synchronization error is significantly reduced, which proves that the Rz torque feed-forward is effective when the Y component moves.

2. X component moves and Y component is stationary.

**Figure 24:**
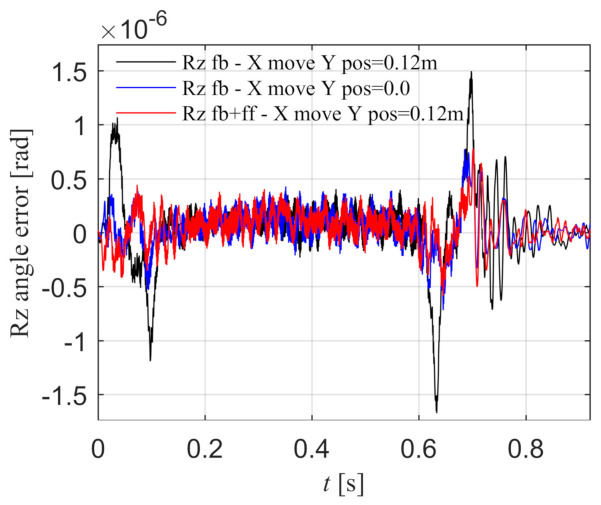
When X component moves and Y component is stationary in one side position of the beam, the Rz angle error with and without feed-forward control in the experiment.

When the Y component is located at one side position of the beam, the Rz angle error under the feedback control is shown as the black line in Figure 24, and the peak error is 1.674 × 10^−6^ rad. When the Y component is stationary in the middle position of the beam, the Rz angle error under the feedback control is shown as the blue line, and the peak error is 7.728 × 10^−7^ rad. It can be seen that when the Y component is not located in the middle position, the X component motion will cause the angle error, and when the Y component is in the middle position, the X component motion will not cause the angle error, which is consistent with the disturbance torque model mentioned above. The red line is the Rz angle error with torque feed-forward control according to Equation (42) when the Y component is located at one side position, and the peak error is 7.777 × 10^−7^ rad. The synchronization error is significantly reduced, which proves that the Rz torque feed-forward is effective when X component moves.

The experimental results show that the proposed synchronization control method based on model decoupling is effective. The multiple-degree-of-freedom decoupled control loops of the platform can meet the given design specifications with the proposed systematic feedback controller design method. The introduction of the fractional biquad filter in the Rz loop is effective to ensure the tracking and disturbance rejection performance of the system simultaneously. Feed-forward control in all loops can reduce the control error. Thus, the precise X and Y position tracking control and the Rz zero rotation control, that is, the synchronous motion control of two motors, are realized in the H-type air floating motion platform.

## 4. Conclusions

This paper proposes a synchronous control method based on the decoupled dynamic model for the H-type air floating motion platform. In the synchronous control loop, a new fractional order biquad filtering method is proposed to adjust the phase of the second-order low damping oscillation system for rotation control of the direction with dual motors, which can ensure the stability and disturbance rejection performance of the system, to realize an accurate synchronous control in the direction with dual motors. A systematic feedback controller design method is proposed to meet the given design specifications in frequency domain, gain crossover frequency, phase margin, and gain margin. A fractional order biquad filter can make the system have lower peak value of the process sensitive transfer function than that with the integer order biquad filter. The comparison results in simulation and experiment demonstrate that the system with fractional order filter has better disturbance rejection performance over that with the traditional filter. The disturbance torque in the Rz caused by the platform motion is modeled, and the effectiveness of the torque feed-forward compensation method is verified in the simulation and experiment, and the synchronization control accuracy is improved significantly.

In future work, the order of the fractional order biquad filter in the Rz loop can be designed as adjustable parameters, so that the system can meet four design specifications, and the corresponding calculation method of control parameters needs to be redesigned.

## Figures and Tables

**Table 1:** The parameters of the motion platform.

Symbol	Value	Description
$m_{x}$	54.90 kg	Mass of X moving component
$m_{y}$	25.05 kg	Mass of Y moving component
m	79.95 kg	Mass of the entire moving component
$J_{Xz}$	7.224 kgm^2^	The rotational inertia of the X component around the Z-axis at its centroid
$J_{Yz}$	0.296 kgm^2^	The rotational inertia of the Y component around the Z-axis at its centroid
$J_{z0}$	7.520 kgm^2^	When the Y component is in the middle of the stroke, the rotational inertia of the entire moving component around the Z-axis at its centroid
$k_{xH}$	30.0 × 10^6^ N/m	Equivalent stiffness of X horizontal air floating guideway
$c_{xH}$	1000 Ns/m	Equivalent damping of X horizontal air floating guideway
$d_{xH}$	0.187 m	The distance between the action points of X horizontal air floating force along X-direction
$k_{yH}$	17.0 × 10^6^ N/m	Equivalent stiffness of Y horizontal air floating guideway
$c_{yH}$	400 Ns/m	Equivalent damping of Y horizontal air floating guideway
$d_{yH}$	0.245 m	The distance between the action points of Y horizontal air floating force along Y-direction
$d_{m}$	1.09 m	The distance between X1 and X2 motors along Y-direction
$d_{r}$	1.012 m	The distance between X1 and X2 reading heads along Y direction
$x_{yc}$	0.0018 m	The distance between the centroid of Y component and the centroid of the entire component along X direction
$x_{xc}$	−0.001 m	The distance between the centroid of X component and the centroid of the entire component along X direction
$x_{F_{y}c}$	−0.024 m	The distance between Y motor and the centroid of the entire component along X direction
$K_{fx_{1}}$	220 N/A	X1 motor thrust constant
$K_{fx_{2}}$	220 N/A	X2 motor thrust constant
$K_{fx}$	220 N/A	X-direction thrust constant
$K_{f\theta }$	220 N/A	Rz thrust constant
$K_{fy}$	230 N/A	Y motor thrust constant
τ	0.0015 s	System delay time
Parameters Varying With the Position of Y Component		
$d_{m1}=\frac{d_{m}}{2}+\frac{m_{y}}{m_{x}+m_{y}}y\left(t\right)$		The distance between X1 motor and the centroid of the entire component along Y direction
$d_{m2}=\frac{d_{m}}{2}-\frac{m_{y}}{m_{x}+m_{y}}y\left(t\right)$		The distance between X2 motor and the centroid of the entire component along Y direction
$d_{r1}=\frac{d_{r}}{2}+\frac{m_{y}}{m_{x}+m_{y}}y\left(t\right)$		The distance between X1 reading head and the centroid of the entire component along Y direction
$d_{r2}=\frac{d_{r}}{2}-\frac{m_{y}}{m_{x}+m_{y}}y\left(t\right)$		The distance between X2 reading head and the centroid of the entire component along Y direction
$y_{xc}=-\frac{m_{y}}{m_{x}+m_{y}}y\left(t\right)$		The distance between the centroid of X component and the centroid of the entire component along Y direction
$y_{yc}=\frac{m_{x}}{m_{x}+m_{y}}y\left(t\right)$		The distance between the centroid of Y component and the centroid of the entire component along Y direction
The Measured Feedback Parameters		
y		Position measured by Y reading head
$x_{1}$		Position measured by X1 reading head
$x_{2}$		Position measured by X2 reading head
$x_{c}$		Position of the centroid of the entire component along X direction
$\theta_{z}$		Angle of the entire component around the Z-axis at its centroid

**Table 2:** Calculation results of the control parameters.

r	$k_{p-\theta }$	$f_{i-\theta }\;(\mathrm{Hz})$	$f_{n1-\mathrm{biq}-\theta }\;(\mathrm{Hz})$	$G_{\mathrm{psm}-\theta }\;(\mathrm{dB})$
1.0	75,545.300	808.683	38.659	−121.430
0.8	324,727.973	199.790	32.481	−127.462
0.7	494,237.255	135.381	32.382	−128.250
0.6	681,282.334	101.793	34.039	−127.392
1.0	75,545.300	808.683	$f_{n1-\mathrm{biq}-\theta }=f_{n-\theta }$	−99.270

**Table 3:** The specification error between the Rz simulated and theoretical open-loop Bode diagrams.

r	Specification	Theoretical Model	Simulated Model	Absolute Error
1.0	Gain crossover frequency	10.254 Hz	10.254 Hz	0.0
	Phase margin	81.799°	81.843°	0.044°
	Gain margin	9.990 dB	10.108 dB	0.118 dB
0.7	Gain crossover frequency	10.254 Hz	10.254 Hz	0.0
	Phase margin	81.855°	82.006°	0.151°
	Gain margin	9.996 dB	10.024 dB	0.028 dB

**Table 4:** The specification error between the X simulated and theoretical open-loop Bode diagrams.

Specification	Theoretical Model	Simulated Model	Absolute Error
Gain crossover frequency	36.115 Hz	36.115 Hz	0.0
Phase margin	39.974°	39.609°	0.365°
Gain margin	10.080 dB	9.957 dB	0.123 dB

**Table 5:** The specification error between the Y simulated and theoretical open-loop Bode diagrams.

Specification	Theoretical Model	Simulated Model	Absolute Error
Gain crossover frequency	36.115 Hz	36.115 Hz	0.0
Phase margin	39.974°	40.003°	0.029°
Gain margin	10.080 dB	9.582 dB	0.498 dB

**Table 6:** The specification error between the Rz simulated and experimental open-loop Bode diagrams.

r	Specification	Theoretical Model	Experimental Model	Absolute Error
1.0	Gain crossover frequency	10.010 Hz	10.010 Hz	0.0
	Phase margin	81.993°	81.454°	0.539°
	Gain margin	9.978 dB	15.476 dB	5.498 dB
0.7	Gain crossover frequency	10.010 Hz	10.010 Hz	0.0
	Phase margin	81.995°	81.569°	0.426°
	Gain margin	9.988 dB	15.503 dB	5.515 dB

**Table 7:** The specification error between the X experimental and theoretical open-loop Bode diagrams.

Specification	Theoretical Model	Experimental Model	Absolute Error
Gain crossover frequency	36.006 Hz	37.349 Hz	1.343 Hz
Phase margin	39.996°	38.169°	1.827°
Gain margin	9.996 dB	9.368 dB	0.628dB

**Table 8:** The specification error between the Y experimental and theoretical open-loop Bode diagrams.

Specification	Theoretical Model	Experimental Model	Absolute Error
Gain crossover frequency	36.006 Hz	35.884 Hz	0.122 Hz
Phase margin	39.996°	39.824°	0.172°
Gain margin	9.996 dB	10.402 dB	0.406 dB

## Data Availability

Date sharing is not applicable.

## Appendix A. X and Y Feedback Controller Design

In X and Y control loop, when the design specifications are gain crossover frequency $f_{c}$, phase margin $\varphi_{m}$, and gain margin $h_{m}$, the open-loop transmission function $G_{o}\left(s\right)=C_{\mathrm{PID}}\left(s\right)\cdot P_{\mathrm{fl}}\left(s\right)$ of the system can meet the following constraints.

1. The phase margin at the gain crossover frequency $f_{c}$ is $\varphi_{m}$.
  (A1) $$∠G_{o}\left(j2\pi f_{c}\right)=∠C_{\mathrm{PID}}\left(j2\pi f_{c}\right)+∠P_{\mathrm{fl}}\left(j2\pi f_{c}\right)=-\pi +\varphi_{m}.$$
2. The amplitude at the gain crossover frequency $f_{c}$ is 1.
  (A2) $$\left|G_{o}\left(j2\pi f_{c}\right)\right|=\left|C_{\mathrm{PID}}\left(j2\pi f_{c}\right)P_{\mathrm{fl}}\left(j2\pi f_{c}\right)\right|=1.$$
3. The phase at the phase crossover frequency $f_{x}$ is −π.
  (A3) $$∠G_{o}\left(j2\pi f_{x}\right)=∠C_{\mathrm{PID}}\left(j2\pi f_{x}\right)+∠P_{\mathrm{fl}}\left(j2\pi f_{x}\right)=-\pi .$$
4. The gain margin at the phase crossover frequency $f_{x}$ is $h_{m}$.
  (A4) $$\left|G_{o}\left(j2\pi f_{x}\right)\right|=\left|C_{\mathrm{PID}}\left(j2\pi f_{x}\right)P_{\mathrm{fl}}\left(j2\pi f_{x}\right)\right|=10^{-\frac{h_{m}}{20}}.$$

The frequency response of $G_{o}\left(s\right)$ is

(A5) $$\begin{array}{ll} G_{o}\left(j2\pi f\right) & =C_{\mathrm{PID}}\left(j2\pi f\right)\cdot P_{\mathrm{fl}}\left(j2\pi f\right)\\ & =k_{p}\left[1+j\left(\frac{f}{f_{d}}-\frac{f_{i}}{f}\right)\right]\frac{1}{-k{\left(2\pi f\right)}^{2}}\frac{f_{n-\mathrm{lp}2}{}^{2}}{f_{n-\mathrm{lp}2}{}^{2}-f^{2}+j2\zeta_{\mathrm{lp}2}f_{n-\mathrm{lp}2}f}e^{-j\tau \left(2\pi f\right)} \end{array}.$$

Its amplitude and phase are

(A6) $$\begin{array}{lll} \left|G_{o}\left(j2\pi f\right)\right| & = & \left|C_{\mathrm{PID}}\left(j2\pi f\right)\right|\cdot \left|P_{\mathrm{fl}}\left(j2\pi f\right)\right|\\ & = & \frac{k_{p}}{f_{d}f}\sqrt{{\left(f_{d}f_{i}-f^{2}\right)}^{2}+{\left(f_{d}f\right)}^{2}}\cdot \\ & & \frac{1}{k{\left(2\pi f\right)}^{2}}\frac{f_{n-\mathrm{lp}2}{}^{2}}{\sqrt{{\left(f_{n-\mathrm{lp}2}{}^{2}-f^{2}\right)}^{2}+{\left(2\zeta_{\mathrm{lp}2}f_{n-\mathrm{lp}2}f\right)}^{2}}} \end{array}.$$

(A7) $$\begin{array}{ll} ∠G_{o}\left(j2\pi f\right) & =∠C_{\mathrm{PID}}\left(j2\pi f\right)+∠P_{\mathrm{fl}}\left(j2\pi f\right)\\ & =\arctan\frac{f^{2}-f_{d}f_{i}}{f_{d}f}-\tau \left(2\pi f\right)-\pi -\arctan\frac{2\zeta_{\mathrm{lp}2}f_{n-\mathrm{lp}2}f}{f_{n-\mathrm{lp}2}{}^{2}-f^{2}} \end{array}.$$

From Equation (A1), we can obtain,

(A8) $$f_{i}=\frac{f_{c}{}^{2}}{f_{d}}-f_{c}\tan\left(\varphi_{m}-A\right),$$

where $A=-\arctan\frac{2\zeta_{\mathrm{lp}2}f_{n-\mathrm{lp}2}f_{c}}{f_{n-\mathrm{lp}2}{}^{2}-f_{c}{}^{2}}-\tau \left(2\pi f_{c}\right)-\pi$.

From Equation (A2), we can obtain,

(A9) $$k_{p}=\frac{f_{d}f_{c}}{B\cdot \sqrt{{\left(f_{d}f_{i}-f_{c}{}^{2}\right)}^{2}+{\left(f_{d}f_{c}\right)}^{2}}},$$

where $B=\frac{1}{k{\left(2\pi f_{c}\right)}^{2}}\frac{f_{n-\mathrm{lp}2}{}^{2}}{\sqrt{{\left(f_{n-\mathrm{lp}2}{}^{2}-f_{c}{}^{2}\right)}^{2}+{\left(2\zeta_{\mathrm{lp}2}f_{n-\mathrm{lp}2}f_{c}\right)}^{2}}}$.

From Equation (A3), we can obtain,

(A10) $$f_{i}=\frac{f_{x}{}^{2}}{f_{d}}-f_{x}\tan\left(D\right),$$

where $D=-\arctan\frac{2\zeta_{\mathrm{lp}2}f_{n-\mathrm{lp}2}f_{x}}{f_{n-\mathrm{lp}2}{}^{2}-f_{x}{}^{2}}-\tau \left(2\pi f_{x}\right)-\pi$.

From Equation (A4), we can obtain,

(A11) $$k_{p}=\frac{f_{d}f_{x}10^{-\frac{h_{m}}{20}}}{E\cdot \sqrt{{\left(f_{d}f_{i}-f_{x}{}^{2}\right)}^{2}+{\left(f_{d}f_{x}\right)}^{2}}},$$

where $E=\frac{k}{k{\left(2\pi f_{x}\right)}^{2}}\frac{f_{n-\mathrm{lp}2}{}^{2}}{\sqrt{{\left(f_{n-\mathrm{lp}2}{}^{2}-f_{x}{}^{2}\right)}^{2}+{\left(2\zeta_{\mathrm{lp}2}f_{n-\mathrm{lp}2}f_{x}\right)}^{2}}}$.

$k_{p}$ can be obtained by substituting Equation (A8) into (A9).

(A12) $$k_{p}=\frac{1}{B\cdot \sqrt{{\left(\tan\left(\varphi_{m}-A\right)\right)}^{2}+1}}.$$

By substituting Equation (A10) into (A11), the equation of $k_{p}$ and $f_{x}$ can be obtained,

(A13) $$k_{p}=\frac{10^{-\frac{h_{m}}{20}}}{E\cdot \sqrt{{\left(\tan\left(D\right)\right)}^{2}+1}}.$$

Since $k_{p}$ is known, then Equation (A13) is an equation about $f_{x}$. Graphical method can be used to find the intersection of Equation (A13) and 0 axis, then $f_{x}$ can be obtained.

By substituting Equation (A8) into (A10), the explicit expression of $f_{d}$ with respect to $f_{x}$ can be obtained,

(A14) $$f_{d}=\frac{f_{c}{}^{2}-f_{x}{}^{2}}{f_{x}\tan\left(D\right)+f_{c}\tan\left(\varphi_{m}-A\right)}.$$

Then $f_{d}$ can be calculated from Equation (A14) and $f_{i}$ can be obtained by substituting $f_{d}$ into Equation (A18). To sum up, $k_{p}$, $f_{i}$, and $f_{d}$ can be determined.
